# The resident gut microbiome modulates the effect of synbiotics on the immunogenicity after SARS-COV-2 vaccination in elderly and diabetes patients

**DOI:** 10.1038/s41522-025-00804-9

**Published:** 2025-08-25

**Authors:** Lin Zhang, Shilan Wang, Martin C. S. Wong, Chris K. P. Mok, Jessica Y. L. Ching, Joyce W. Y. Mak, Chunke Chen, Bing Huo, Shuai Yan, Chun Pan Cheung, Emily O. L. Chiu, Emily Y. T. Fung, Pui Kuan Cheong, Francis K. L. Chan, Siew C. Ng

**Affiliations:** 1 Microbiota I-Center (MagIC), The Chinese University of Hong Kong, Hong Kong SAR, China; 2 New Cornerstone Science Laboratory, The Chinese University of Hong Kong, Hong Kong SAR, China; 3 Department of Anaesthesia and Intensive Care, Faculty of Medicine, The Chinese University of Hong Kong, Hong Kong SAR, China; 4 Li Ka Shing Institute of Health Sciences, State Key Laboratory of Digestive Disease, Institute of Digestive Disease, The Chinese University of Hong Kong, Hong Kong SAR, China; 5 Jockey Club School of Public Health and Primary Care, Faculty of Medicine, The Chinese University of Hong Kong, Hong Kong SAR, China; 6 Centre for Health Education and Health Promotion, Faculty of Medicine, The Chinese University of Hong Kong, Hong Kong SAR, China; 7 SH Ho Research Centre for Infectious Diseases, Faculty of Medicine, The Chinese University of Hong Kong, Hong Kong SAR, China; 8 School of Biomedical Sciences, The Chinese University of Hong Kong, Shatin, New Territories, Hong Kong SAR, China; 9 Department of Medicine and Therapeutics, Faculty of Medicine, The Chinese University of Hong Kong, Hong Kong SAR, China; 10 Centre for Gut Microbiota Research, Faculty of Medicine, The Chinese University of Hong Kong, Hong Kong SAR, China; 11 The D.H. Chen Foundation Hub of Advanced Technology for Child Health (HATCH), The Chinese University of Hong Kong, Hong Kong SAR, China

**Keywords:** Metagenomics, Microbial ecology, Microbiome, Metagenomics

## Abstract

The study aims to tackle the seed and soil microbiome and mechanisms that contribute to the effect of synbiotics in enhancing immunogenicity after SARS-CoV-2 vaccination in elderly and diabetic patients. Among 369 subjects who received 3 months of SIM01, a gut microbiota-derived synbiotic formula of three *Bifidobacterium* strains (*B. adolescentis*, *B. bididum*, and *B. longum*) or a placebo after the SARS-CoV-2 vaccines (mRNA vaccine BNT162b2 (Pfizer-BioNTech) or the inactivated vaccine Sinovac-CoronaVac), we performed metagenomic sequencing in stool samples of 280 vaccinees collected at baseline and 3-month postvaccination and metabonomic sequencing in 276 vaccinees collected at baseline and 1-month postvaccination. The open niche of autochthonous gut microbiota (lower levels of *Bifidobacterium* and decreased functional potential for carbohydrate metabolism) was associated with enhancing SIM01-contained species. The enrichment of three *bifidobacterial* species after 3 months of SIM01 intervention (BABBBL_fc) was positively correlated with the level of neutralizing antibodies to the BNT162b2 vaccine at 6-month postvaccination. The fold change of benzoic acid was positively correlated with BABBBL_fc in the BNT162b2 vaccinees, which was also implicated with SARS-CoV-2 surrogate virus neutralization test (sVNT)% levels at 1-month postvaccination. Importantly, SIM01 strain engraftment assessed by StrainPhlAn (A metagenomic strain-level population genomics tool) was associated with a higher fold change of three *bifidobacterial* species and could be predicted based on the baseline gut microbiome. Therefore, the resident gut microbiome affected the SIM01 engraftment, which was associated with the immunogenicity of SARS-CoV-2 BNT162b2 vaccines.

## Introduction

Vaccines for SARS-CoV-2 have demonstrated efficacy in preventing infection and mitigating disease severity; however, the determinants influencing their immunogenicity and duration of protection are not yet fully understood. The elderly and individuals with diabetes mellitus were at an increased risk of more severe manifestations and higher mortality rates from COVID-19^1–6^. Also, the antibody responses following COVID-19 vaccination in diabetes patients and the elderly were diminished^7–10^. Aging is associated with declining innate and adaptive immune system functionality^11^. In diabetic patients, immune responses are compromised due to the negative impacts of insulin deficiency and hyperglycemia on cellular immunity^12^. Hence, exploring strategies that enhance postvaccination immunogenicity in these vulnerable groups is imperative.

The gut microbiota is crucial for maintaining the host’s health by performing key functions, including nutrient provision, vitamin synthesis, facilitation of metabolic processes, and modulation of the immune system^13^. Our prior observation studies of the gut microbiota in recipients of COVID-19 vaccines demonstrated that postvaccination immune response enhancement or sustainability is associated with certain microbial markers or metabolites^14,15^. In addition, our previous study suggested that SIM01 hastened antibody formation against SARS-CoV-2, reduced nasopharyngeal viral load, reduced pro-inflammatory immune markers, and restored gut dysbiosis in hospitalized COVID-19 patients^16^, and also alleviated multiple symptoms of Post-acute COVID-19 syndrome (PACS)^17^. These previous findings suggest using interventions such as gut microbial derivatives to modulate post-vaccine immune responses. However, it remains to be determined whether microbiome modulation with SIM01 could serve as an effective intervention to optimize immune responses following vaccination.

Probiotics are known to modulate the immune system through various mechanisms^18,19^. However, probiotic engraftment has various challenges, with successful, long-lasting engraftment being intricately associated with the host’s autochthonous gut microbiota. There is nutritional competition between probiotics and the autochthonous gut microbiota, such as competition for carbon sources following niche overlap^20^. An investigation on one probiotic strain, *Bifidobacterium longum* AH1206, demonstrated that gut microbiota composition and the absence of specific functional capabilities—particularly the deficiency in genes of carbohydrate utilization—are principal determinants for the probiotic long-term colonization^21^. There is a lack of extensive prospective investigation specifically targeting high-risk populations to examine the rehabilitation of intestinal microbial ecology with multiple probiotic strains and what determines probiotic colonization.

Based on the Immune Microbiome Product Against COVID infecTion (IMPACT) study, we conducted a post hoc analysis of IMPACT to investigate (1) the impact of the SIM01 probiotic formulation on the gut microbiota and metabolites and how key elements that contribute to the probiotics engraftment, (2) how gut microbiome and metabolites modulated the immunogenicity of COVID-19 vaccines after SIM01 intake in elderly recipients and those with type 2 diabetes. This research aimed to enhance our understanding of how to optimize the immunogenicity in vulnerable populations following vaccination based on the resident microbiome^22^.

## Results

### The gut microbiome of subjects receiving SIM01 was more likely to transition towards *Bifidobacterium* enriched-cluster II

**Fig. 1:**
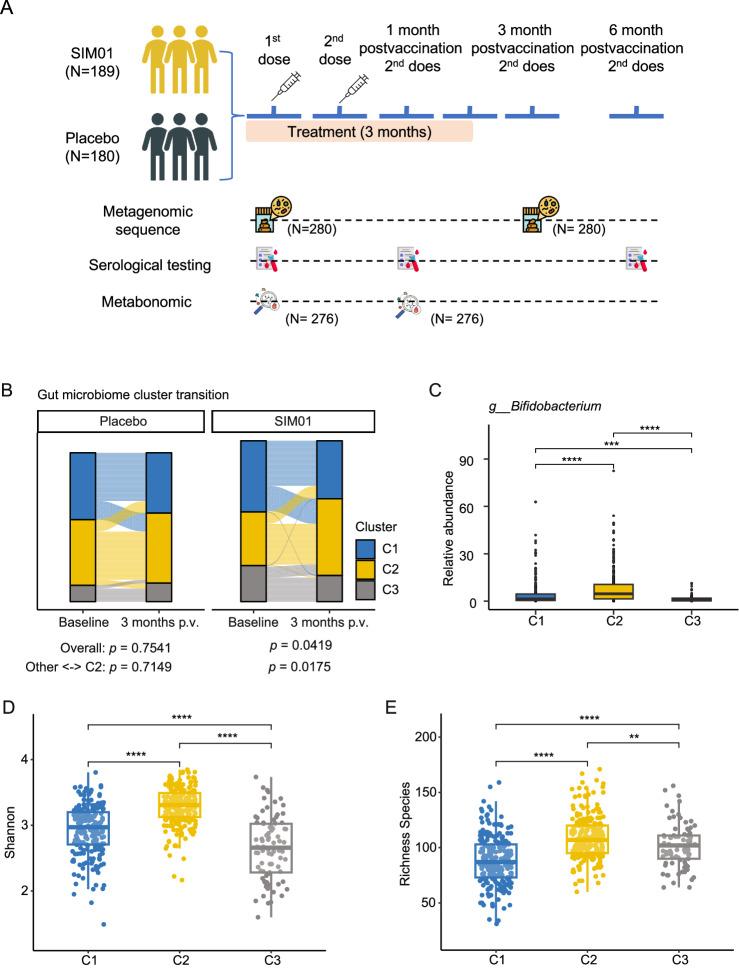
Study design and change in gut community cluster from baseline to 3-month after the second vaccine dose (postvaccination, p.v.) after 3 months of SIM01 intervention. **A** Study design. Eligible subjects received a gut microbiota-derived synbiotic formula (SIM01) or placebo for 3 months in all subjects aged ≥65 years or with type 2 diabetes mellitus (T2DM). All subjects received the COVID-19 vaccine, including either the mRNA vaccine BNT162b2 (Pfizer-BioNTech) or the inactivated vaccine Sinovac-CoronaVac, within 1 week of intervention. Study visits were arranged at 1-month, 3-month, and 6-month after completion of the second dose of the COVID-19 vaccine. **B** The Sanky diagram shows the transition of three gut community clusters between baseline and 3-month postvaccination (p.v.) following a 3-months SIM01 intervention in both placebo and SIM01 groups. The width of each flow corresponds to the number of subjects undergoing an intra- or inter-cluster transition. The height of each bar is proportional to the number of subjects in each group. *p* values were given by the Chi-square test. The first line of *p* values indicated the overall comparison (transition in three clusters before and after treatment). The second line of *p* values indicates the subgroup comparison transition between cluster 2 and other clusters (combined C1 and C3). C cluster. **C** The relative abundance of the *Bifidobacterium* genus in the samples with distinct gut community clusters. *p* values were given by the Wilcoxon test (two-sided). **D**, **E** The gut microbiome Shannon diversity (**D**) and the richness of observed species (**E**) in the samples with distinct gut community clusters. *p* values were given by the Wilcoxon test (two-sided). Elements on boxplots: center line, median; box limits, upper and lower quartiles; whiskers, 1.5×IQR; points, samples. IQR interquartile range. * indicated *p* < 0.05, ** indicated *p* < 0.01, *** indicated *p* < 0.001, **** indicated *p* < 0.0001 after adjustment for multiple comparisons.

From April 2021 to March 2022, a total of 369 SARS-CoV-2 vaccinees (51.49% females; median age: 67.00 years old) received either SIM01 (189 participants) or placebo (180 participants), and available stool samples were included in this study. There was no significant difference in baseline characteristics between the placebo and SIM01 groups among BNT162b2 vaccinees or CoronaVac vaccinees (Table S1). Study visits were arranged at 1 month, 3 months, and 6 months after completion of the second dose of the COVID-19 vaccine. Shotgun metagenomic sequencing was performed to assess gut microbiota composition in stool samples at baseline and 3 months after the second vaccine dose (postvaccination). Blood samples at 1-month or 6-month postvaccination were subjected to the SARS-CoV-2 surrogate virus neutralization test (sVNT) and spike receptor-binding domain (RBD) IgG ELISA testing. Metabonomic sequencing was performed to assess the gut metabolite profile in fresh stool samples at baseline and 1-month postvaccination (Fig. 1A).

The enterotypes (clusters) are useful for describing the gut microbial community structure and are linked to health and disease^23^. Therefore, to investigate the impact of SIM01 on the gut microbiome composition, we checked the gut microbiome cluster at baseline and 3-month postvaccination. The microbial community cluster at the genus-level was performed using the partitioning around medoid (PAM) method, including all samples from the SIM01 and placebo groups. Three clusters were identified, including cluster I dominated by the *Bacteroides* genus, cluster II enriched by *Eubecterium* and *Bifidobacterium* genus, and cluster III dominated by the *Prevotella* genus (Fig. 1B and Fig. S1A, B), which aligns with the existing enterotype classification^23^. Overall, SIM01 affected the gut microbiome cluster change before and after treatment (Chi-squared test, *p* = 0.0419, Fig. 1B), but Placebo did not (Chi-squared test, *p* = 0.07541). Further, we sub-grouped all samples into cluster 2 and other clusters (combined clusters 1 and 3). Indeed, subjects receiving SIM01 were more likely to transition to cluster II (Chi-squared test, *p* = 0.0175, Fig. 1B), which was characterized by a higher relative abundance of *Bifidobacterium* (Fig. 1C) and higher gut microbiome diversity and richness (Fig. 1D, E). Of note, the T2DM subjects had lower gut two *Bifidobacterium* species (*B. longum* and *B. pseudocatenulatum*) at baseline (Fig. S1C), while they had significantly higher *B.bifidum* after 3 months of SIM01 intervention after adjusting for age and sex (Fig. S1D).

### The autochthonous gut microbiota affects the enhancement of SIM01-contained species

Then, we were interested in whether baseline features could affect the enhancement of SIM01-contained species within the SIM01 arm. Interestingly, subjects from cluster I/III at baseline had a significantly enriched SIM01 species at 3-month postvaccination compared to control, but not subjects from cluster II at baseline (Fig. S2A). This result was also confirmed in three individual *Bifidobacterium* species (Fig. S2B, C, D).

**Fig. 2:**
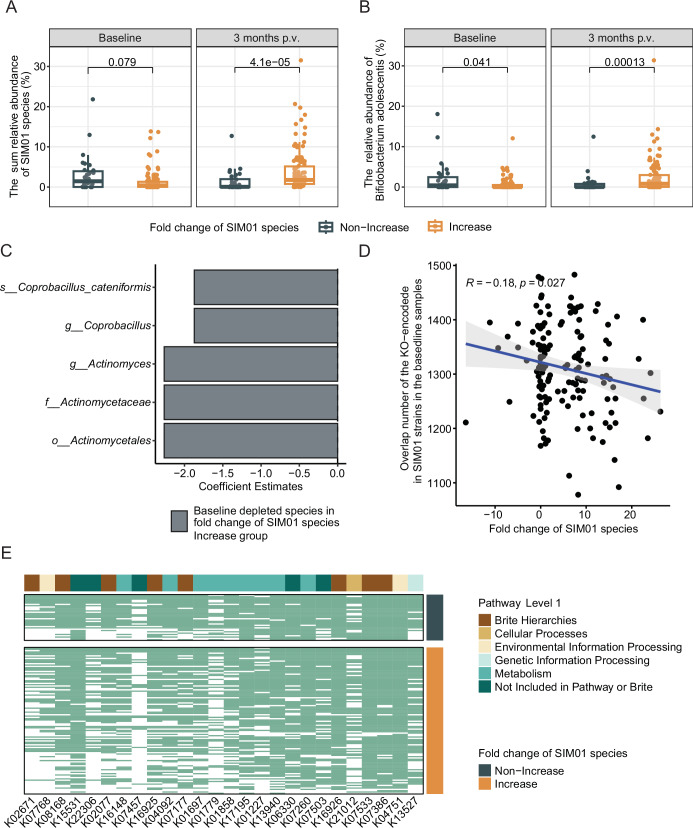
The autochthonous gut microbiota affects the enhancement of SIM01-contained species. **A**, **B** The changes in the sum relative abundance of three *Bifidobacterium* (**A**) or *B. adolescentis* (**B**) between baseline and 3-month postvaccination (p.v.) after subgrouping based on the increase or non-increase of SIM01-contained species within the SIM01 arm. Non-Increase, *N* = 34 each time point. Increase, *N* = 110 at each time point. **C** The depleted species at baseline in the gut of subjects who were characterized by an increase in SIM01-contained species after 3 months of treatment. Maaslin2 was used for identification after adjusting the gender, age, and T2D status. *p* < 0.05, FDR <0.25. **D** Correlation between fold change of SIM01 species and the overlap number of the KO-encoded in SIM01 strains in the baseline samples. Coefficients and *p* values of the correlations were given by Spearman’s correlation tests. **E** The heatmap showed the presence and absence of baseline KOs that were associated with the enhancement of SIM01-related species after 3 months of SIM01 intervention. Each row represents the baseline sample of each subject.

Further, we grouped the subjects within the SIM01 arm according to the fold change of relative abundance of SIM01 species (Increase group: fold change >0, Non-increase group: fold change ≤0, see method details). In particular, the increased group had a lower *B. adolescentis* at baseline than the non-increase group (Fig. 2A, B), and the increased group did have a higher relative abundance of SIM01 species collectively or separately at 3-month postvaccination (Fig. 2A, B and Fig. S2E, F). Besides, the depletion of baseline bacteria from the *Actinomyces* genus of the Actinobacteria phylum and the *Coprobacillus* genus was linked to the enrichment of species contained in SIM01 (Fig. 2C). Moreover, we checked the overlap number between the KEGG Orthology (KO) detected in the baseline samples and the KO encoded by SIM01 strains. Correlation results indicated that the overlap KO number is negatively associated with the fold change of SIM01 species (Fig. 2D, *R* = −0.18, *p* = 0.027), which indicates that the functional overlap may play a role in the baseline ecological effect on the SIM01 enhancement, but it is still affected by the incompleteness of the current microbiome function database. This result was also repeated in three individual species (Fig. S2G). Further, we compared the BABBBL_fc between baseline samples with or without specific KO. Specifically, the lack of 26 KOs (out of 857) at baseline was associated with the enhancement of SIM01-contained species (Fig. 2E and Table S2, FDR <0.05), especially KOs of metabolism class (carbohydrate metabolism, glycan biosynthesis and metabolism, amino acid metabolism). In summary, when the allochthonous species colonize a new community, their colonization outcome can vary depending on the resident gut microbiome.

### The enhancement of the SIM01 species is associated with SARS-CoV-2 vaccine immunogenicity

**Fig. 3:**
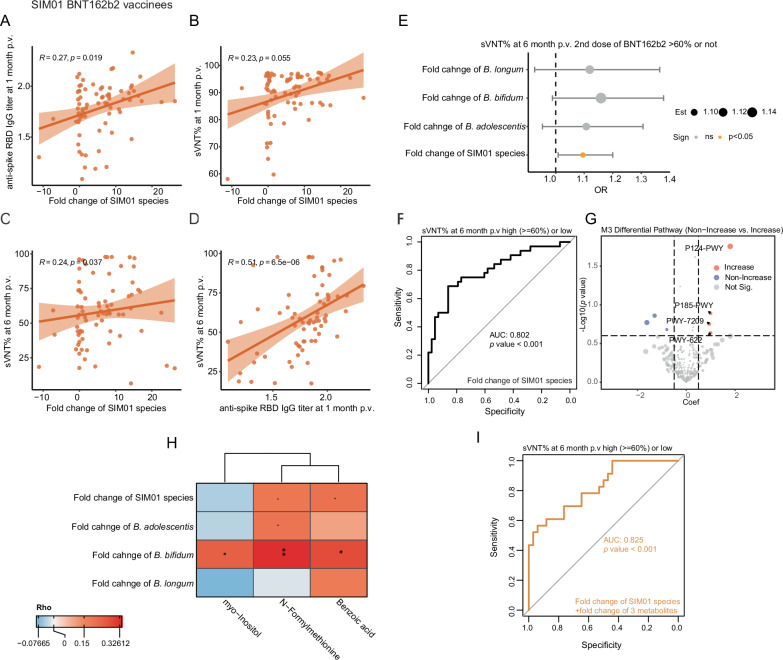
SIM01-induced *Bifidobacterium* increase in the gut after 3 months of treatment is associated with higher neutralizing antibodies at 6 months after BioNTech mRNA vaccination. **A**, **B** The correlation between the fold change of three *Bifidobacterium* in the gut after 3 months of SIM01 treatment and the anti-spike receptor-binding domain (RBD) (**A**) or the neutralizing antibody sVNT (%) (**B**) at 1 month after the second dose of BioNTech was examined using Spearman’s correlation test. **C** The correlation between the fold change of three *Bifidobacterium* in the gut after 3 months of SIM01 treatment and the neutralizing antibody sVNT (%) at 6 months after the second dose of BioNTech was examined using Spearman’s correlation test. *N* = 75. **D** The correlation between the anti-spike receptor-binding domain (RBD) at 1 month after the second dose of BioNTech and the neutralizing antibody sVNT (%) at 6 months after the second dose of BioNTech was examined using Spearman’s correlation test. Regression lines with 95% CI (orange area) were shown on scatter plots. **E** Logistic regression based on fold change of individual *Bifidobacterium species* or a combined model based on fold change of all three *Bifidobacterium species* adjusting age, gender, and T2D status was used to calculate the odds ratio (OR, 95% Confidence interval) values for the for high (≥60%, *n* = 32) vs. low sVNT levels (<60%, *n* = 43) responders among BioNTech (BNT162b2) vaccines at 6 month after second dose within SIM01 arm. Each OR was presented as a dot with a bar showing the 95% CI. *p* < 0.05 was presented as an orange dot. OR, odds ratio; T2D, type 2 diabetes mellitus; fc fold change. **F** AUROC (95% CI) values of models based on combined fold change of three species contained in SIM01 for high (≥60%) vs. low sVNT levels (<60%) at 6-month postvaccination adjusting age, gender, and T2D status. p.v., postvaccination. **G** The volcano plot shows the differential pathway between two groups of subjects with the non-increase or increase SIM01 species in the gut after 3 months of treatment within the SIM01 arm. Maaslin2 was used for identification after adjusting the gender, age, and T2D status. *p* < 0.05, FDR < 0.25. **H** Correlations between the fold change of the relative abundance of SIM01-contained three species and the fold change of differential metabolites between baseline and 1-month postvaccination in BioNTech vaccinees. The label within each frame: · indicated *p* < 0.1, * indicated *p* < 0.05, ** indicated *p* < 0.01 based on Spearman correlation analysis. The color indicates the correlation Rho value. **I** AUROC (95% CI) values of models based on the combined fold change of three species contained in SIM01 and the fold change of three metabolites between baseline and 1-month postvaccination for high (≥60%) vs. low sVNT levels (<60%) at 6-month postvaccination, adjusting age, gender, and T2D status. p.v. postvaccination, T2D type 2 diabetes mellitus.

Next, we would like to test our hypothesis on whether the enhancement of the SIM01 species could enhance SARS-CoV-2 vaccine immunogenicity. Considering that the immune response varies among different types of vaccines^24^, the following analysis will subgroup according to vaccine type (Table S3). Interestingly, the fold change of SIM01 species was positively correlated with the anti-spike RBD IgG titer at 1-month postvaccination BNT162b2 in the SIM01 arm (*R* = 0.27, *p* = 0.019, Fig. 3A) and positively correlated with sVNT% level at 1-month postvaccination BNT162b2 in the SIM01 arm, but not statistically significant (*R* = 0.23, *p* = 0.055, Fig. 3B).

Considering the SARS-CoV-2 infectious and booster dose of vaccine will affect the immune response^24^, the subjects infected by SARS-CoV-2 after 3-month postvaccination and before 6-month postvaccination were excluded from the following analysis (*n* = 19), and whether subjects received a booster dose at 6-month postvaccination was taken into consideration. Notably, the enhancement of SIM01-contained species was positively correlated with sVNT% level at 6-month postvaccination of the second dose of BNT162b2 in the SIM01 arm (Fig. 3C), as well as subjects who received the third dose of BNT162b2 vaccination 6-month postvaccination (Fig. S3A). After subgrouping according to the elderly and type 2 diabetes population, a similar correlation was found in elderly subjects after the second dose of BNT162b2 vaccination, but not in the subjects who received the 3^rd^ dose of BNT162b2 vaccination, which may be related to the limited sample size after subgrouping (Fig. S3B). Besides, the spike in RBD IgG titer at 1-month postvaccination was also positively associated with sVNT% 6-month postvaccination BNT162b2 in the SIM01 arm (Fig. 3D). Importantly, enhancement of SIM01 species could predict sVNT% levels at 6-month postvaccination (>60% or not, which corresponded to twice 50% protection titer^25^) after the second dose of BNT162b2 vaccination within the SIM01 arm, together with age, sex, and T2DM (*p* = 0.0378, Fig. 3E). Together with age, sex, and T2DM, enhancement of SIM01 species could predict sVNT% levels at 6-month postvaccination after the second dose of BNT162b2 vaccination within the SIM01 arm with an AUC = 0.802 (*p* < 0.001, Fig. 3F). Further, we compared the gut microbial functional pathway at 3-month postvaccination between Increase and Non-increase groups based on fold change of relative abundance of SIM01 species within the SIM01 group that received BNT162b2, and four pathways enriched in the SIM01-contained species-increase group, namely *Bifidobacterium* shunt (P124-PWY), formaldehyde assimilation III dihydroxyacetone cycle (P185-PWY), super pathway of pyrimidine ribonucleosides degradation (PWY-7209), and starch biosynthesis (PWY-622) (Fig. 3G). However, the above patterns were not found in the CoronaVac group (Table S3), which may be due to the limited sample size in the CoronaVac group and the different mechanisms of action of inactivated and mRNA vaccines. Also, similar results were not found in the placebo arm that received BNT162b2 (Table S3), which indicated that the enhancement of SIM01-contained species after intervention correlated with the SARS-CoV-2 vaccine immunogenicity, but not endogenous enrichment of three *Bifidobacterium* species.

### The metabolite change induced by the SIM01 treatment improves the prediction for SARS-CoV-2 vaccine immunogenicity

We further detected the metabolites of available fresh stool samples collected at baseline (*n* = 276) and 1-month postvaccination (*n* = 276). A total of 338 metabolites were detected, and 290 metabolites with prevalence >25% were applied for further analysis. First, we separately identified the differential metabolites between baseline and 1-month postvaccination after adjusting for vaccine types within the two arms. There were 24 metabolites that were significantly changed from baseline to 1-month postvaccination after the SIM01 treatment (Table S4), with six overlapping with the placebo group (Fig. S3C and Table S5).

To identify the gut metabolites related to the SIM01 intervention, we further checked the correlation between the fold change of 18 unique metabolites in the SIM01 group (Fig. S3C) and the fold change of the relative abundance of SIM01-contained three species (BABBBL_fc). For CoronaVac vaccinees, one metabolite (Taurocyamine) was identified as positively correlated, and four metabolites (Myo-inositol, L-sorbose, pyridoxal, and ricinoleic acid) were identified as negatively correlated with the BABBBL_fc or fold change of the relative abundance of any SIM01-contained species (Table S6). Notably, three metabolites (*N*-formylmethionine, benzoic acid, myo-inositol) were identified as positively correlated with the BABBBL_fc or fold change of the relative abundance of any SIM01-contained species in the BNT162b2 vaccinee (Fig. 3H and Table S6). Among them, the fold change of Benzoic acid between baseline and 1-month postvaccination is positively correlated with sVNT% levels at 1-month postvaccination (*R* = 0.2274, *p* value = 0.048, Table S7). Although the fold change of these three metabolites between baseline and 1-month postvaccination is not significantly correlated with sVNT% levels at 6-month postvaccination, adding the fold change of these three metabolites in the prediction model could improve the AUC = 0.825 of predicted sVNT% levels at 6-month postvaccination (>60% or not, which corresponded to twice 50% protection titer^25^) after the second dose of BNT162b2 vaccination within the SIM01 arm (*p* < 0.001, Fig. 3I). Specifically, benzoic acid ranked in the top 1 among these three metabolites in the prediction model, and a previous study also showed that several *Bifidobacteria* species were able to convert albiflorin to benzoic acid in vitro^26^.

### The engraftment of the SIM01 strain could be predicted by the baseline gut microbiome

**Fig. 4:**
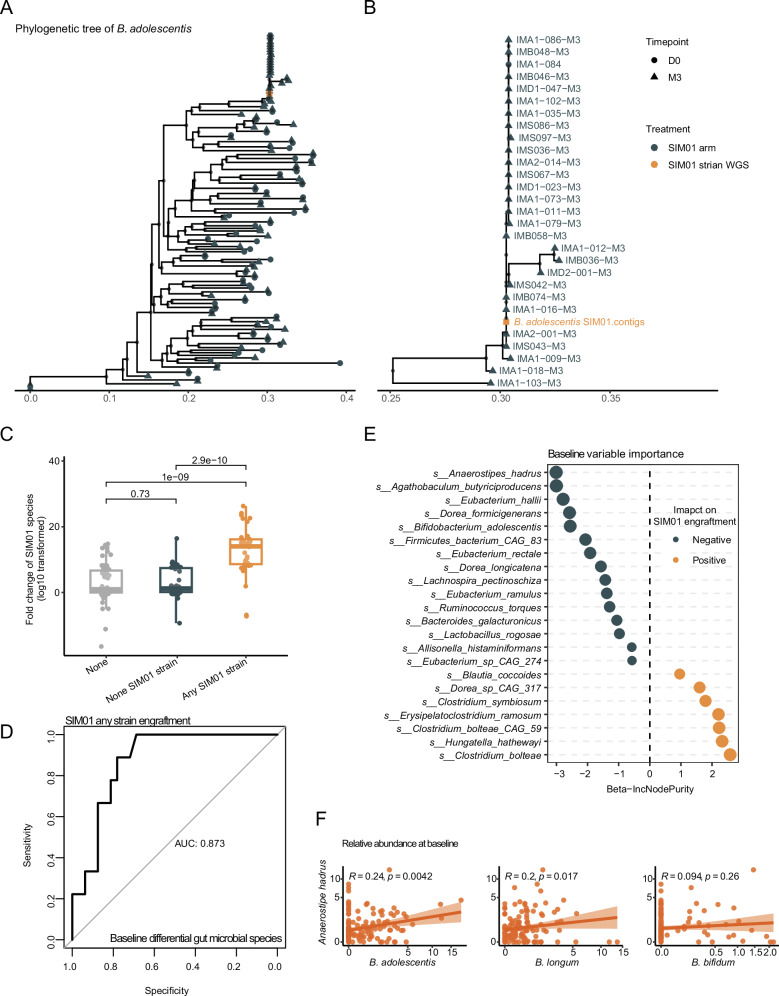
The engraftment of the SIM01 strain based on StrainPhlAn. **A** Phylogenetic tree of dominant haplotypes of *B. adolescentis* per sample. **B** Subset of the phylogenetic tree of dominant haplotypes of *B. adolescentis* of the samples with SIM01 *B. adolescentis* strain as the dominant strain after 3 months of treatment. Orange circles and text represent the reference genomes of the SIM01 *B. adolescentis* strain. **C** The fold change of relative abundance of SIM01-contained three species in three groups of subjects (None: without any *Bifidobacterium* species contained in SIM01 that involved in the StrainPhlAn construction, None SIM01 strain: with *Bifidobacterium* species involved in the StrainPhlAn construction but not the same as the SIM01 strain, Any SIM01 strain: with at least one *Bifidobacterium* strain contained in SIM01 that involved in the StrainPhlAn construction). *p* values were given by the Wilcoxon test (two-sided). Points, samples. Elements on boxplots: center line, median; box limits, upper and lower quartiles; whiskers, 1.5×IQR. **D** AUROC (95% CI) values of models about SIM01 strain engraftment based on baseline differential gut microbial species. **E** The variable importance of features in the prediction model. **F** Correlation between the relative abundance of baseline SIM01-contained three *Bifidobacterium* species with the relative abundance of *Anaerostipes hadrus*. Coefficients and *p* values of the correlations were given by Spearman’s correlation tests.

Colonization is key for probiotics to exert sufficient host interaction to confer health benefits. We used StrainPhlAn to evaluate whether the SIM01 strain became dominant after 3 months of intervention based on the pairwise genetic distance (Fig. S4A, see Methods for details). *B. adolescentis* had the highest engraftment rate (28/144 subjects (19.44%), Fig. 4A, B), followed by *B. longum* (12/144 subjects (8.33%)) and *B. bifidum* (7/144 subjects (4.86%), Fig. S4B, C), which echoed the abundance of *Bifidobacterium* species in the adult gut reported previously^27^. The subjects with any SIM01 strain that successfully became the dominant strain were consistently characterized by significantly higher fold change of three SIM01-contained species (*p* < 0.01, Fig. 4C). Also, the benzoic acid was significantly higher in BNT162b2 vaccinees with any SIM01 strain than those with non-SIM01 bifidobacteria species (Fig. S5A).

To precisely predict the SIM01 colonization based on the baseline microbial community, we first used Maaslin2 to identify the species associated with the engraftment of any SIM01 strain (Table S8). Based on the 22 species identified, the random forest was employed for the classification prediction model with an AUC = 0.873 (Fig. 4D). The variable importance is shown in Fig. 4E, with the baseline *B. adolescentis* ranked fifth with a negative impact on the SIM01 strain colonization. The top one species that negatively impacted the SIM01 strain colonization is *Anaerostipes hadrus*, which indicated the availability of *Bifidobacterium* species at baseline (Fig. 4F). *Anaerostipes spp*., butyrate-producers, could be cross-fed by the metabolites of *Bifidobacterium* spp in the infant's gut^28^. High relative abundance of baseline gut *Clostridium bolteae*, *Clostridium bolteae CAG 59*, and *Clostridium symbiosum* was associated with any SIM01 strain colonization. Therefore, the colonization potential of probiotics has been attributed to strain properties and is more permissive to certain individuals, which is implicated with the baseline microbial community.

## Discussion

This research investigated the impact of the SIM01, a gut microbiota-derived synbiotic formula, on the gut microbiota and metabolites, as well as its correlation with the immunogenicity of COVID-19 vaccines in elderly recipients and those with type 2 diabetes. The study identified a correlation between baseline levels of specific gut bacterial species and the engraftment of SIM01-contained strains, implying that the intrinsic characteristics of the host’s autochthonous gut microbiota could influence the colonization efficacy of SIM01. Furthermore, the research suggested the augmentation of vaccine immunogenicity predicated on the gut microbial community profile and metabolites, offering novel insights for personalized vaccine adjuvant selection and the optimization of vaccine efficacy in vulnerable populations.

The influence of the autochthonous gut microbiota on the colonization of introduced bacterial strains is evidenced by (1) the subjects with baseline gut microbiome cluster II (high relative abundance *Bifidobacterium* genus) were implicated with lower enhancement of SIM01-contained species, (2) the association between lower levels of *B. adolescentis* and less carbohydrate metabolism function in the resident microbiome and enrichment of SIM01 species, (3) the predictable engraftment of SIM01 strain based on baseline gut microbiome. Specifically, *Anaerostipes spp*., butyrate-producers and could be cross-fed by the metabolites of *Bifidobacterium* spp. in infants’ gut^28^, were positively correlated with the resident *Bifidobacterium* spp., which might indicate an occupied niche (resource requirement overlap between allochthonous probiotic and autochthonous microbiota is high)^29^. This finding aligns with a previous study where lower pretreatment levels of *Bifidobacterium longum* in the gut microbiota were predictive of the *B. longum* AH1206 persistence^21^. Gut-resident bacteria adapt to specific niches by engaging in intricate competitive or cooperative interactions with other microbes^30,31^. These interactions regulate microbial community structure and restrict colonization by alien microbes^32^. For instance, sequential administration of identical bacterial strains to germ-free mice results in the first strain occupying the niche exclusively, while the subsequent strain cannot establish itself^33^. As stated in “The Origin of Species“^34^, it posits that introduced species have a greater likelihood of thriving in environments devoid of their close phylogenetic relatives. Besides, the association between high relative abundance of gut baseline *Clostridium bolteae, C. symbiosum*, and SIM01 later colonization may reflect metabolic synergy, as these species produce acetate^35^ and modulate bile acids^36^, which may facilitate engraftment. However, as *C. bolteae* is also implicated in dysbiosis, its role may depend on the ecological context and might reflect the vulnerability of the population^37^. Taken together, the diversity of the autochthonous gut microbiota (resident gut microbiome) can predict the colonization success of the allochthonous probiotic strain, which helps us better understand the relative niche and fitness difference.

The sVNT% level is a parameter commonly employed to assess the efficacy of vaccine-induced immune response. This study utilized the fold change of baseline and 3-month postvaccination SIM01 species to predict the sVNT level at 6-month postvaccination, which indicates the potential adjuvant effect of synbiotics on immune responses. Consistently, previous studies showed that *Bifidobacterium* species can influence postvaccination immune responses. In elderly individuals with a mean age of 86.7 years, *Bifidobacterium longum* BB536 appears to augment innate immunity, as its continuous intake before and after influenza vaccination correlated with reduced fever and influenza rates postvaccination^38^. In infants, *Bifidobacterium longum* subspecies *infantis* has been associated with improved humoral and T cell responses to Bacillus Calmette–Guérin, polio, and tetanus vaccines^39^. Animal models also indicate that *Bifidobacterium animalis* ssp. *lactis* (BB-12) boosts immune responses following seasonal influenza vaccination^40^.

Compared to the placebo group, subjects receiving SIM01 were more likely to transition to cluster II from baseline to 3-month postvaccination. Notably, in the placebo group, the endogenous enrichment of three *Bifidobacterium* species induced by the placebo did not correlate with the sVNT level at 6-month postvaccination, suggesting that the host had developed immune tolerance to the *Bifidobacterium* species. In contrast, the allochthonous enrichment of three *Bifidobacterium* species included by SIM01 was more effective in subjects who were not characterized by cluster II (which displays a high relative abundance of the *Bifidobacterium* genus) at baseline, and thus, the immune response facilitated the immunogenicity after elderly and diabetes patients received BNT162b2 vaccination.

Compared with the gut microbial pathways of the Non-increase group based on the fold change of relative abundance of SIM01 species at 3-month postvaccination, starch biosynthesis (PWY-622) was enriched in the SIM01 species-increase group, which may benefit other probiotics’ growth. According to an in vitro study, *B. adolescentis* 22 L has cross-feeding relationships with *B. bifidum* PRL2010, which promotes the growth and survival rate of the latter, possibly through sugars released from starch degradation^41^. In addition, *B. bifidum* PRL2010 specifically works for the extracellular breakdown of host-glycans that will again support the growth of other members of the *Bifidobacterium* bacterial community through simple sugars^42^. These may indicate that SIM01 partially plays a role that relies on interactions between bacterial species. Gut microbiota could exert diverse effects on immune function through direct interaction with immune cells in the gut and indirect mechanisms such as secreting metabolites and regulating the systemic availability of critical metabolites^43^. Notably, we identified that the fold change of benzoic acid (positively correlated with the fold change of SIM01-contained species) between baseline and 1-month postvaccination is also positively correlated with the sVNT% levels at 1-month postvaccination. Several *Bifidobacteria* species were able to convert albiflorin to benzoic acid in vitro based on a previous report^26^. And gut bacteria-derived benzoic acid can be absorbed into the bloodstream from the gut^44^. A previous study showed that benzoic acid enhances the intestinal expression of the polymeric immunoglobulin receptor, B-cell activating factor from the TNF family, and activation-induced cytidine deaminase (AID), thereby enhancing IgA production by B-cells in chickens^45^. Benzoic acid derivatives have been reported to possess anti-influenza virus activities and immune-modulatory effects^46^. The evidence underscores the potential of benzoic acid to bolster immune functions and partially explains why it is relevant to vaccine immune response. Therefore, the SIM01 may modulate the immune response through direct interaction with the gut community and indirect mechanisms such as secreting metabolites.

There are several strengths in our study. First, this study highlights the unique SARS-CoV-2 vaccinee characteristics of the population in Hong Kong, China. It examines participants who received either inactivated vaccines or mRNA-based SARS-CoV-2 vaccines, while mainland China’s predominant use of inactivated SARS-CoV-2 vaccines^47^. Secondly, the randomized controlled trial setting facilitates understanding of the enhancement of endogenous and allochthonous *Bifidobacterium* species. Thirdly, the whole-genome sequencing of three SIM01 Strains and the metagenomic sequence of stool samples collected from the subjects who received SIM01 for 3 months allowed us to check the engraftment of probiotics with multiple strains.

However, there are also some limitations. First, COVID-19 infection along the subject’s follow-up makes it hard to track long-term vaccine-induced-sVNT, which restricts the understanding of the prolonged effects of SIM01. As we know, vaccine antibody levels decline over time. Admittedly, during the COVID-19 pandemic, achieving perfect synchronization between gut microbiome sampling (stool collection) and immune profiling (blood sampling) was challenging. In our study, our results showed that the fold change of SIM01 species was positively correlated with both the immune index at 1-month postvaccination BNT162b2 and 6-month postvaccination of the 2nd dose of BNT162b2, which covered the time point when the gut metagenomic sequence was performed. Secondly, due to the deficiency of fresh stool at 3-month postvaccination, we can only detect the gut metabolites at 1-month postvaccination as a proxy to reflect the metabolism profile change induced by SIM01 treatment, but still, we found some metabolites change corresponding to *Bifidobacterium* species that with biological meaning. Besides, it would be hard to conclude that probiotic colonization depends on functional redundancy in the autochthonous gut microbiota based on a bioinformatic analysis only, since the complexities of microbiome functionality and the current limitations in existing pathway databases^48^. It is worthwhile to test out specific strains in vitro, ex vivo, and *vivo* in further studies, such as using co-culture systems to assess how different strains of probiotics interact with existing gut microbiota under controlled conditions (with or without strain utilized the specific carbohydrates e.g. oligosaccharides)^49^ and conducting studies using animal models to evaluate how the existing microbiota affect colonize of different probiotic strains^50^. In addition, previous studies showed that *Bifidobacteria* intervention could improve other types of vaccine immune response, such as the Bacillus Calmette–Guérin vaccine^51^ and 13-valent pneumococcal conjugate vaccine^52^. Therefore, further animal experimental and in vitro studies are necessary to establish causality and the mechanism between the *Bifidobacteria* or metabolites and enhanced COVID-19 vaccine immunogenicity. Last but not least, the lack of different population generalizations is another limitation; to address this, it would be beneficial to establish multiple testing sites across various regions, enabling the observation of microbial variations in a diverse racial demographic. Such an approach would enhance the generalizability of the findings and support further validation.

In summary, compared to the endogenous enrichment of three *Bifidobacterium* species induced by the placebo, the allochthonous enrichment of three *Bifidobacterium* species included by the 3 months treatment of SIM01 was associated with elevated gut benzoic acid levels and the immunogenicity of SARS-CoV-2 BNT162b2 vaccines. Besides, SIM01 engraftment was influenced by baseline gut microbiota. These findings underscore the importance of probiotics as personalized vaccine adjuvant selection and the optimization of vaccine efficacy in vulnerable populations.

## Methods

### Study design, participants

This study was part of a randomized clinical trial^53^, where the primary outcome was a composite of adverse health outcomes. Eligible subjects received a gut microbiota-derived synbiotic formula (SIM01) or placebo for 3 months in all subjects aged ≥65 years or with type 2 diabetes mellitus (T2DM). Subjects were randomized at the time of COVID-19 vaccination to reduce the confounding effects of vaccination on health outcomes. All subjects received the COVID-19 vaccine, including either the mRNA vaccine BNT162b2 (Pfizer-BioNTech) or the inactivated vaccine Sinovac-CoronaVac, within one week of intervention. Study visits were arranged at 1-month, 3-month, and 6-month after completion of the second dose of the COVID-19 vaccine (Fig. 1A). SIM01 is an oral microencapsulated formulation of three lyophilized *Bifidobacteria* at a dose of 20 billion colony-forming units (CFU) per day and 3 prebiotics, including galactooligosaccharides, xylooligosaccharide, and resistant dextrin, as derived from our previous study^54,55^. The three *Bifidobacterium* strains (*B. adolescentis*; *B. bididum*, and *B. longum*) are commercially available from Chambio Co., Ltd. and WECARE-PROBIOTICS. SIM01 has been approved by health authorities and is a patent-protected formula.

Further, a total of 376 subjects with available stool samples were screened out. Based on the inclusion criteria (e.g., without COVID-19 infection before 3-month postvaccination, received SARS-CoV-2 vaccine), 369 participants (SIM01, *N* = 189, Placebo, *N* = 180) were finally included in the study. Among them, 280 participants (SIM01, *N* = 144, Placebo, *N* = 136) with 560 stool samples with stool nucleic acid preservatives (Norgen, Cat#28330) were collected at both baseline and 3-month post the second dose of vaccine were applied for the metagenomic sequence. Besides, 276 participants (SIM01, *N* = 144, Placebo, *N* = 132) with 552 fresh stool samples collected at both baseline and 1-month post the second dose of vaccine were applied for the metabonomic sequence. This study was performed in the general population of Hong Kong from April 2021 to March 2022. The study was approved by the Clinical Research Ethics Committee of the Chinese University of Hong Kong on 19 April 2021 (Ref. No. 2021.186). It was registered on ClinicalTrials.gov (NCT04884776). This study was conducted according to the Declaration of Helsinki and ICH-GCP principles. All subjects provided informed consent.

### Stool metagenomic sequencing and taxonomy, and function profile

Fecal samples were collected at baseline and 3-month postvaccination. We performed metagenomic profiling on fecal samples as previously described.^15,53^ Briefly, fecal DNA was extracted using Maxwell® RSC PureFood GMO and Authentication Kit (Promega, Madison, Wisconsin) following the manufacturer’s instructions. Sequencing libraries were prepared from extracted DNA using the Illumina DNA Prep kit, and sequenced with a paired-end 150 bp sequencing strategy by Illumina NovaSeq 6000 System. The raw sequence data generated 5.945 Gb [5.312-6.946] (median [IQR]) sequence depth per sample. Further, raw sequence reads were filtered and quality-trimmed using Trimmomatic v0.39 and decontaminated against the human genome (Reference: hg37dec_v0) by Kneaddata (V. 0.10.0, https://bitbucket.org/biobakery/kneaddata/wiki/Home). MetaPhlAn3 (v 3.0.13) was used to generate a species-level abundance table. To annotate the function of gut microbiome genes, we applied HUMAnN3 (3.0.0) to all metagenomic samples after filtering out reads mapping to the human reference. Moreover, the UniRef gene families that were detected by HUMAnN3 were mapped to KEGG Orthogroups (KOs) using the humann_regroup_table function, and the abundances of KOs were normalized using the humann_renorm_table function.

### Stool metabolomic sequencing

Fresh stool metabolomes were profiled by liquid chromatography-tandem mass spectrometry (LC-MS/MS) using Waters UPLC I-Class Plus (Waters, USA) equipped with QTRAP 6500 Plus (SCIEX, USA). A high-throughput targeted quantification for a metabolites panel (HM700 Metabolome) can realize the absolute quantification of more than 700 metabolite small molecules, including amino acids, organic acids, fatty acids, sugars, bile acids, carnitine, phenyl or benzyl derivatives, indoles, etc.

### Partitioning around the medoid for the microbial cluster

The microbial community cluster at the genus-level was performed according to a published protocol (https://enterotype.embl.de/enterotypes.html) with the partitioning around medoid (PAM) method, including samples from SIM01 and placebo groups. The optimal number of clusters was chosen based on the Calinski–Harabasz (CH) Index, which shows the performance in recovering the number of clusters.

### The KEGG ortholog (KO) annotation of the SIM01 strain

The whole-genome sequencing (WGS) of three SIM01 strains was performed. Bacterial genomes were assembled using MEGAHIT (v1.2.9)^56^. Prodigal (V2.6.3) was used to predict reliable protein-coding genes^57^. Further, the protein profiling was searched against the curated KOs database using KOfamscan^58^.

### Blood samples sVNT and measurements

Blood samples at baseline, 1-month postvaccination or 6-month postvaccination were subjected to a SARS-CoV-2 surrogate virus neutralization test (sVNT) (GenScript, NJ, USA, Catalog No. L00847-A). Spike receptor-binding domain (RBD) IgG ELISA was used to assess antibody levels in plasma collected at baseline and 1-month postvaccination and carried out as previously described^59,60^. sVNT data was expressed as percentage inhibition (%).

### Strain engagement check use StrainPhlAn

The SIM01 strain engagement check was based on StrainPhlAn and followed the instructions (https://github.com/biobakery/MetaPhlAn/wiki/Strain-Sharing-Inference). Briefly, the genomes of three SIM01 strains obtained via whole-genome sequencing and metagenomic samples of the SIM01 arm were employed for the StrainPhlAn phylogenetic tree construction and strain-level profiling. The pairwise phylogenetic normalized genetic distance (nGD) distribution of the same or different individuals was checked. Youden’s index was used to set the species-specific strain identity thresholds. The nGD between the SIM01 strain and the metagenomic samples at 3-month postvaccination within each species was compared with the corresponding thresholds to decide whether it was a successful engraftment in the gut microbiome. The “tree_subset” was used to subset a tree by the node of the SIM01 strain and return all related nodes within a selected number of levels.

### Statistical analysis

Continuous variables were expressed in median (±interquartile range, IQR), while categorical variables were presented as percentages. Changes in continuous variables, including the quantities of bacteria, were compared by the Wilcoxon rank-sum test, whereas changes in categorical variables were compared using the Chi-square test or Fisher’s exact test. A two-sided *p* value of <5% was considered statistically significant. Alpha microbial diversity was studied using the observer species richness index and the Shannon diversity index based on the species-level profile for each sample. Maaslin2 was used to identify the baseline species associated with T2DM, age, and sex. The significance threshold for these analyses was a false discovery rate (FDR)-corrected *p* value (*q* value) of 0.25.

The fold change of the relative abundance of SIM01-contained three species was calculated based on the sum fold change of the relative abundance of SIM01-contained three individual species.

Fold change = Log10 ^(Relative abundance of one species at 3-month postvaccination/Relative abundance of one species at baseline)^

Fold change of SIM01 species = Fold change (*Bifidobacterium adolescentis*) + Fold change (*Bifidobacterium bifidum*) + Fold change (*Bifidobacterium longum*)

Maaslin2 was used to identify the baseline features or 3-month postvaccination functional pathway associated with the fold change of SIM01 species (Increase group: fold change >0, Non-increase group: fold change ≤0). The correlations were analysed using Spearman’s correlation tests. In the analysis of the metabolites, only metabolites present in more than 25% of samples were included. Maaslin2 was used to identify the differential metabolites between baseline and 1-month postvaccination after adjusting the vaccine type within the two arms, separately. The fold change of metabolites between baselines and 1-month postvaccination was calculated similarly to the fold change of the relative abundance of SIM01-contained three individual species. The logistic regression was used to predict whether the fold change of SIM01 species (or add the fold change of identified metabolites) could classify sVNT(%) at 6-month postvaccination second dose of BNT162b2 > 60% or not with generalized linear regression modeling (GLM) together with age, sex, and T2DM. The area under the ROC Curve (AUC) was obtained via pROC.

Random forest was employed to classify any engraftment of the SIM01 strain. Each data frame was separated into a 70% testing set and a 30% training set. And performed five times cross-validation and repeated ten times in the random forest model. The default parameters of “mtry” were adopted, and the number of trees was 500. The “set.seed” was 100 to ensure reproducibility of results. The area under the receiver operating characteristic curve (AUROC) was calculated to check the model’s performance. The top important variables were defined based on the beta-IncNodepurity from the random forest model (v.4.7-1.1). False discovery rate (FDR) correction was applied for multiple tests. Analyses where FDR correction was not applicable included binary analysis of demographics, GLM, correlations between fold change of SIM01 species (or fold change of metabolites) and immune markers, and Wilcoxon’s tests of features' relative abundance. FDR correction was not applicable because these analyses did not include multiple testing, were based on a priori hypotheses, or had several metrics (multivariable and univariable tests) for marker identification.

All microbiome-related statistical tests were performed with R Statistics version 4.1.3 with the following packages: phyloseq, vegan, tidyverse, dplyr, ggplot2, Maaslin2, random Forest, pROC, and ggpubr.

## Supplementary information

Supplementary Information

## Data Availability

Quality-controlled and human DNA-removed sequence data have been deposited into the NCBI Sequence Read Archive database under BioProjects PRJNA1181778. According to our institutional data sharing policy, requests for data sharing can be submitted with a written proposal as a part of a collaboration effort. The proposal should detail the intended use of the data. The data management team composed of scientists and clinicians will review these requests based on scientific merit and ethical considerations, including patient consent, to avoid any misuse or misinterpretation. Data sharing will be undertaken if the proposed projects have a sound scientific rationale or potential patient benefit. Data recipients are required to enter formal data sharing agreement, which describes the conditions for release and requirements for data transfer, storage, archiving, publication, and intellectual property. Since the data management meeting is held monthly, please anticipate a response within two working months.

## Code availability

This paper does not generate new code.
