# Supplementary material for: The resident gut microbiome modulates the effect of synbiotics on the immunogenicity after SARS-COV-2 vaccination in elderly and diabetes patients
Source: NPJ Biofilms Microbiomes. 2025 Aug 25;11:171. doi: 10.1038/s41522-025-00804-9 (PMC12375777; doi:10.1038/s41522-025-00804-9)
Supplement: Supplementary file 1 — Supplementary Information [file 41522_2025_804_MOESM1_ESM.pdf]

Supplementary information

The resident gut microbiome modulates the effect of synbiotics on the immunogenicity after SARS-COV-2 vaccination in elderly and diabetes patients

Lin Zhang[#], Shilan Wang[#], Martin C.S. Wong, Chris K.P. Mok, Jessica Y.L. Ching, Joyce W.Y. Mak, Chunke Chen, Bing Huo, Shuai Yan, Chun Pan Cheung, Emily O.L. Chiu, Emily Y.T. Fung, Pui Kuan Cheong, Francis K.L. Chan[*], Siew C. Ng[*]

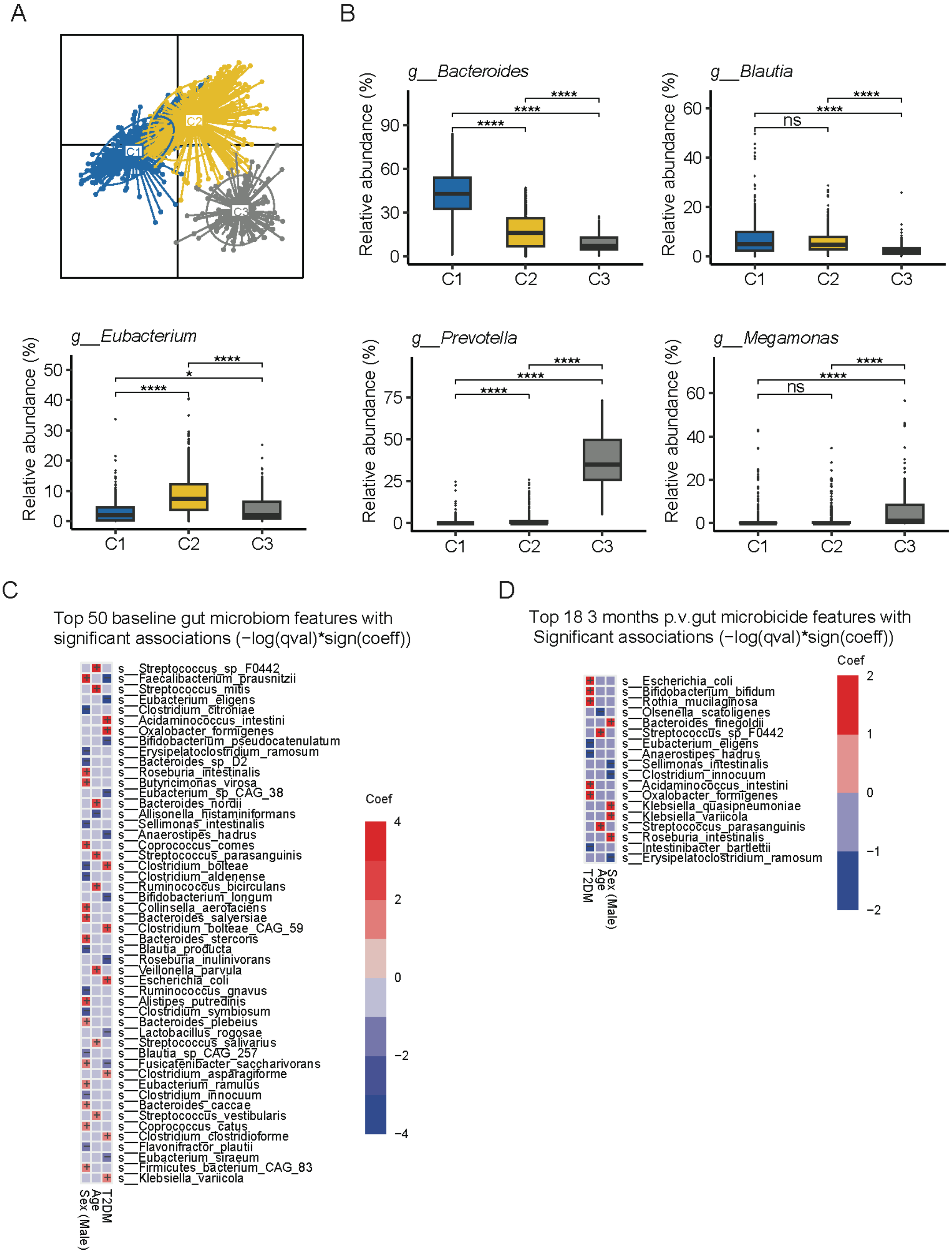

Figure S1: Gut microbiome community cluster. Related to Figure 1.

(A) Gut microbiome community clustering using partitioning around medoid (PAM) procedure.

(B) Relative abundance of top 6 genera in three clusters. P values were given by the Wilcoxon rank-sum test (two-sided). Elements on boxplots: center line, median; box limits, upper and lower quartiles; whiskers, 1.5×IQR; Points, outliers.

(C) Baseline differential gut microbiome of aging and T2D subjects after adjusting the gender identified by Maaslin2. $P < 0.05$, FDR < 0.25.

(D) The differential gut microbiome of aging and T2D subjects at 3-month postvaccine after adjusting the gender identified by Maaslin2. $P < 0.05$, FDR < 0.25.

T2DM, type 2 diabetes mellitus.

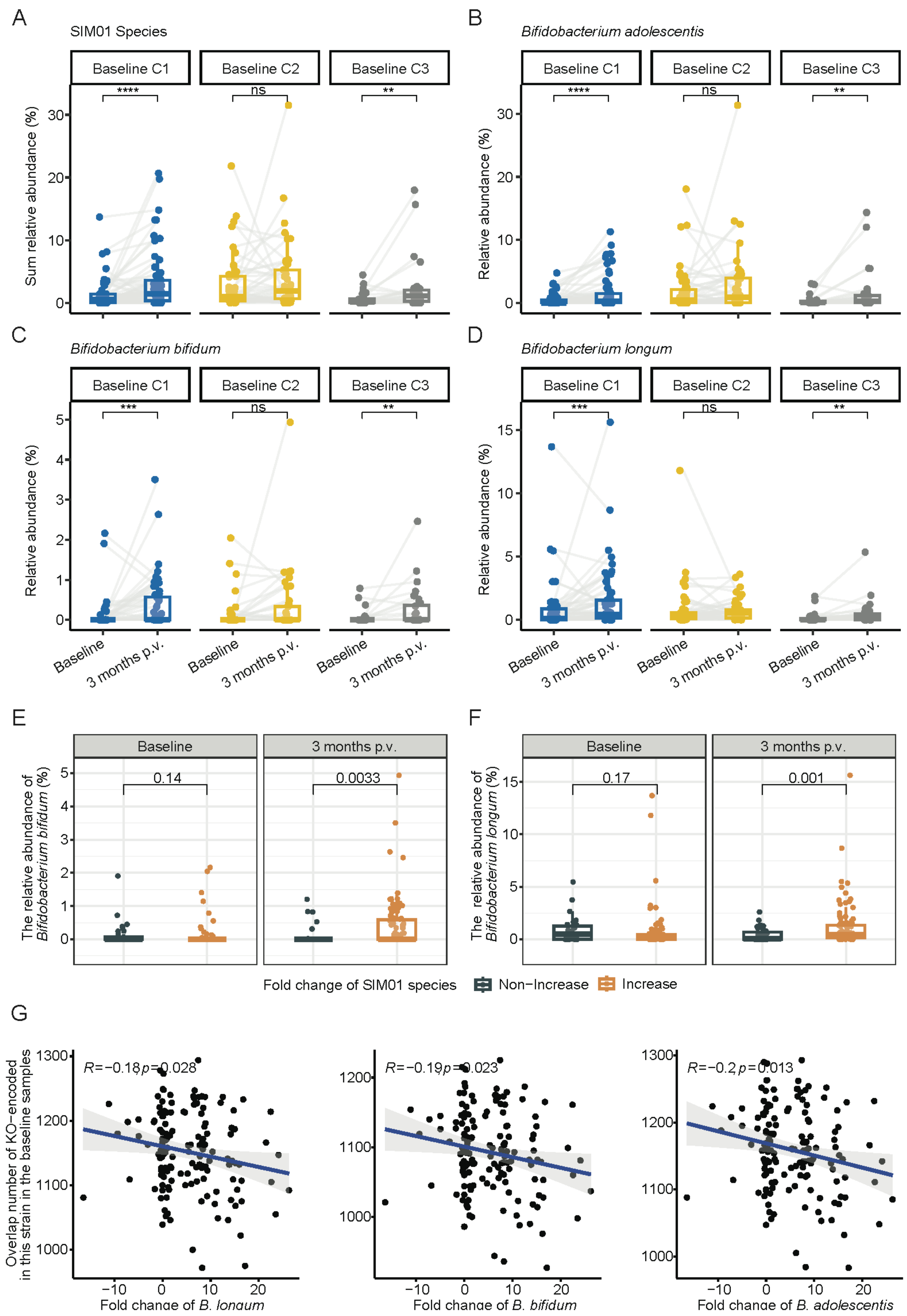

Figure S2: The indigenous gut microbiota affects the enhancement of SIM01-contained species. Related to Figure 2.

(A-D) The changes in the relative abundance of SIM01 contained three species between baseline and 3-month postvaccination after subgrouping based on the baseline gut microbiome cluster within the SIM01 group. The ns indicated non-significant, * indicated $P < 0.05$, ** indicated $P < 0.01$, *** indicated $P < 0.001$, **** indicated $P < 0.0001$

(E-F) The changes of relative abundance of three *B. bifidum (E) and B. longum (F)* between baseline and 3-month postvaccination after subgrouping based on the increase or non-increase of SIM01 contained species.

(G) Correlation between fold change of SIM01 three species individually and overlap number of the KO-encoded in SIM01 strains in the baseline samples. Coefficients and *p* values of the correlations were given by Spearman's correlation tests.

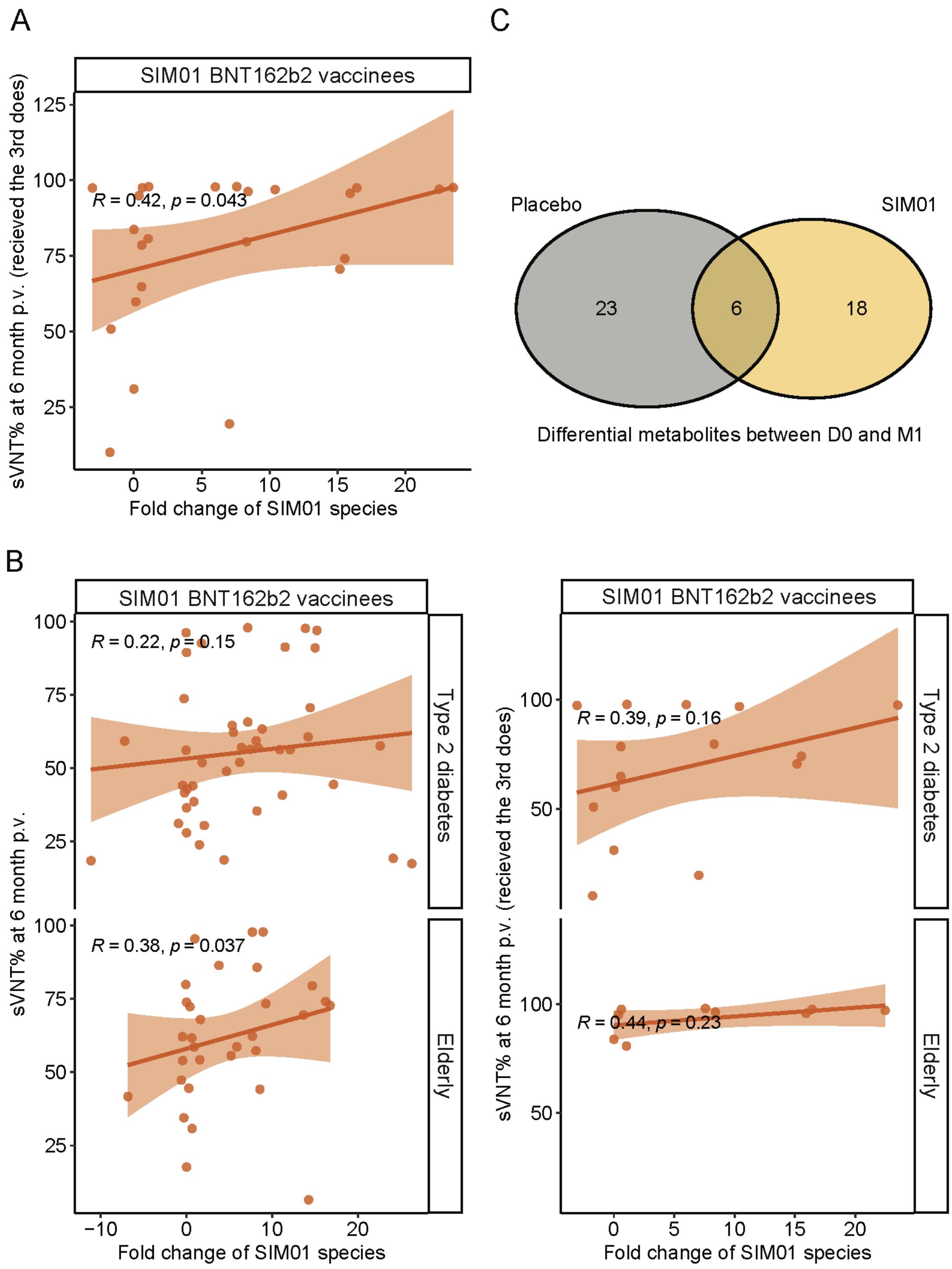

Figure S3. (A) The correlation between the fold change of three *Bifidobacterium* in the gut after 3-month of SIM01 treatment and the neutralizing antibody sVNT(%) at 6-month after 3rd dose of BioNTech was examined using Spearman's correlation test. Regression lines with 95% CI (orange area) were shown on scatter plots. N = 24. Related to Figure 3.

(B) The correlation between the fold change of three *Bifidobacterium* in the gut after 3 months of SIM01 treatment and the neutralizing antibody sVNT (%) at 6-month after 2nd dose of BioNTech (or received the 3rd dose) was examined using Spearman's correlation test after subgrouping according to elderly and type 2 diabetes.

(C) The number of overlap differential gut metabolites between baseline and 1-month postvaccination between the placebo and SIM01 arm.

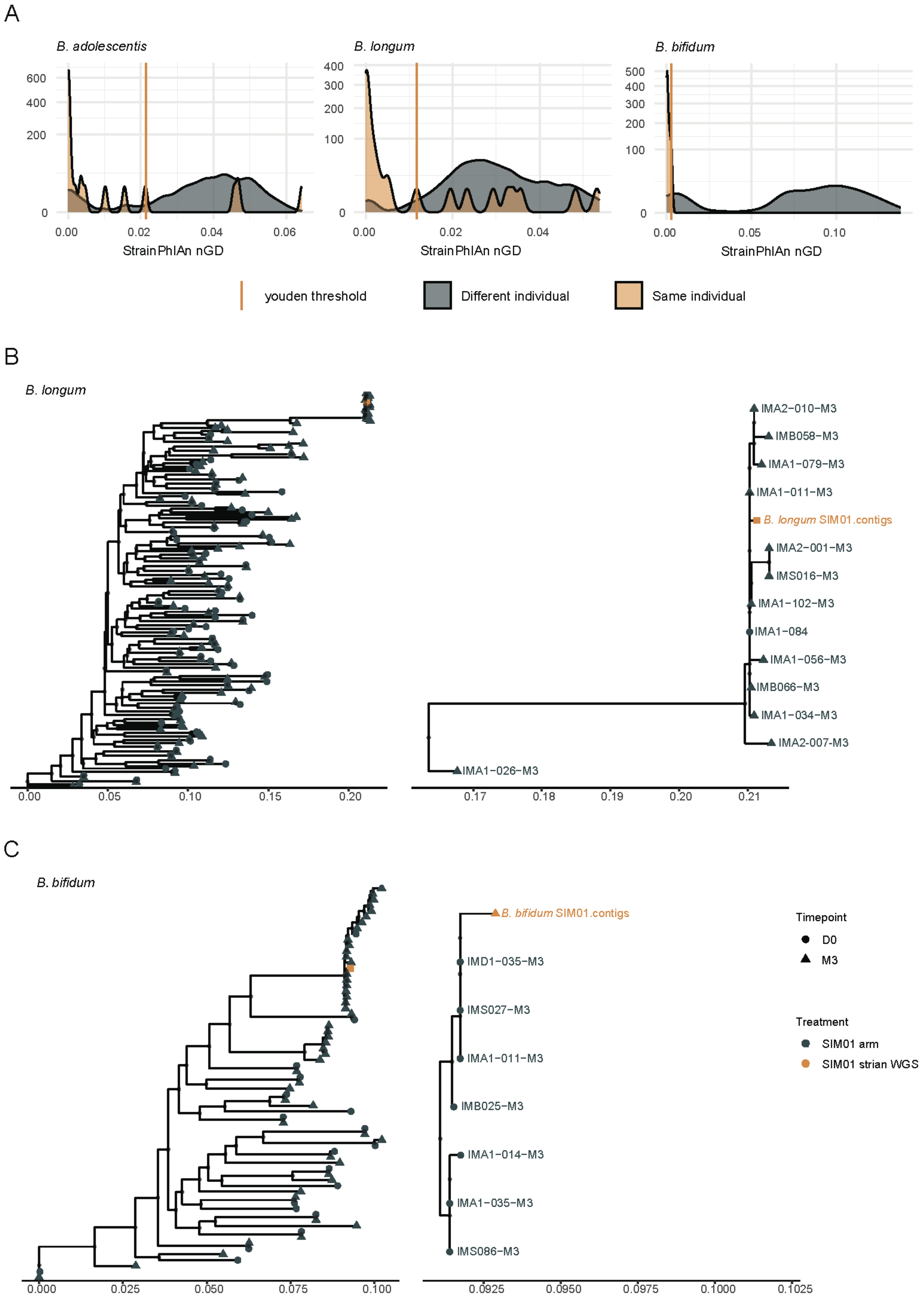

Figure S4. The engraftment of SIM01 strain on vaccine immunogenicity. Related to Figure 4.

(A) The pairwise phylogenetic normalized genetic distance (nGD) distribution of the same or different individuals. Youden's index was used to set the species-specific strain identity thresholds.

(B) Phylogenetic tree of dominant haplotypes of *B. longum* per sample (left). Subset of the phylogenetic tree of dominant haplotypes of *B. longum* of the samples with SIM01 *B. longum* strain as the dominant strain after 3 months of treatment (right). Orange circles and text represent the reference genomes of the SIM01 *B. longum* strain.

(C) Phylogenetic tree of dominant haplotypes of *B. bifidum* per sample (left). Subset of the phylogenetic tree of dominant haplotypes of *B. bifidum* of the samples with SIM01 *B. bifidum* strain as the dominant strain after 3 months of treatment (right). Orange circles and text represent the reference genomes of the SIM01 *B. bifidum* strain.

WGS, Whole-Genome Sequencing

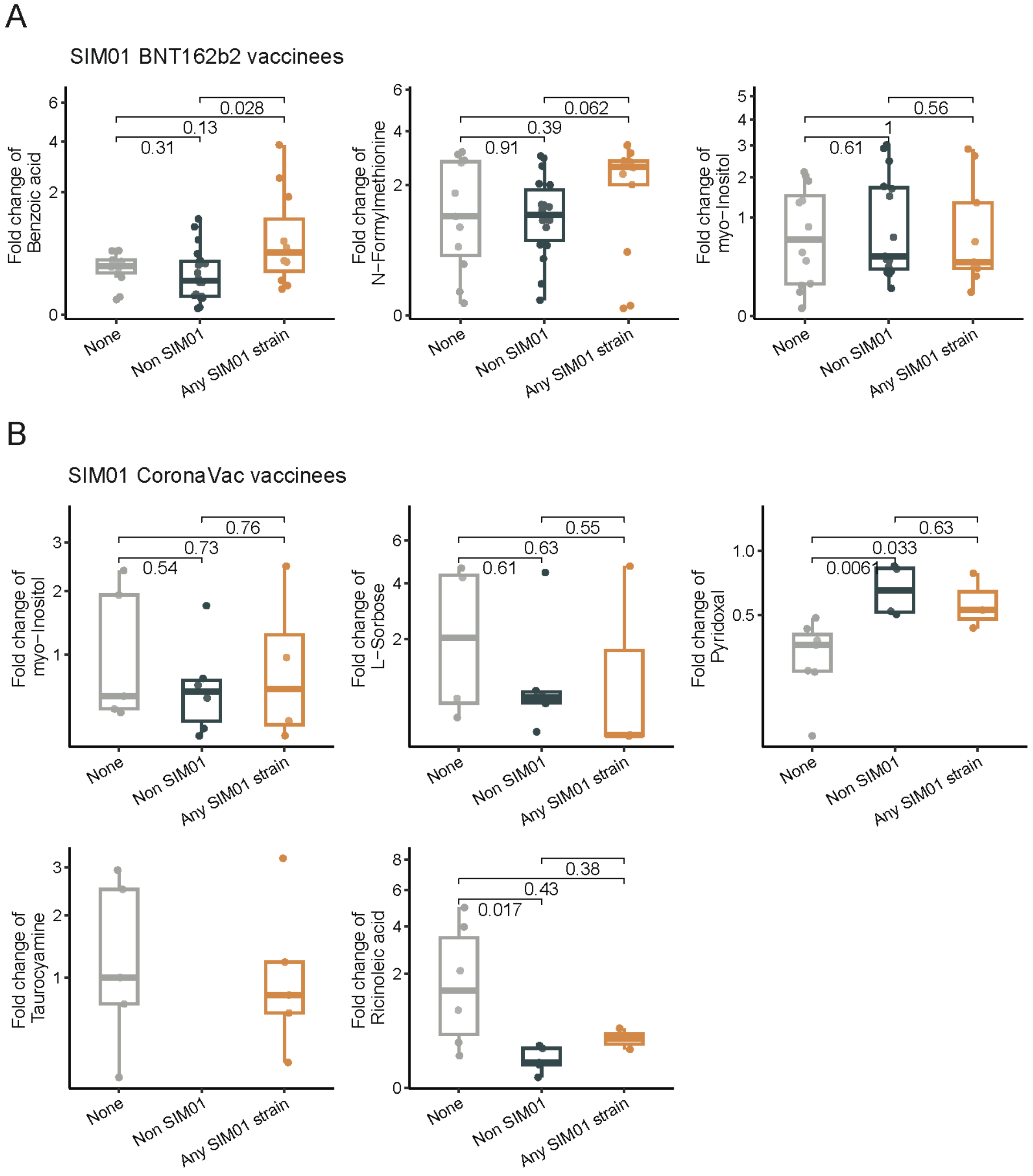

Figure S5. The fold change of identified metabolites between baseline and 1-month postvaccination in three groups of subjects (None: without any *Bifidobacterium* species contained in SIM01 that involved in the Strainphlan construction, None SIM01 strain: with *Bifidobacterium* species involved in the Strainphlan construction but not the same as the SIM01 strain, Any SIM01 strain: with at least one *Bifidobacterium* strain contained in SIM01 that involved in the Strainphlan construction). *P* values were given by the Wilcoxon test (two-sided). Points, samples. Elements on boxplots: center line, median; box limits, upper and lower quartiles; whiskers, 1.5×IQR.

Table S1. Demographic characteristics of the study population.

	BioNTech, N = 220			Sinovac, N = 149		
Characteristic	Placebo, N = 105[1]	SIM01, N = 115[1]	p-value[2]	Placebo, N = 75[1]	SIM01, N = 74[1]	p-value[2]
Female	57 (54%)	55 (48%)	0.3	40 (53%)	38 (51%)	0.8
Age, years	68 (65, 72)	66 (62, 70)	0.2	67 (61, 71)	67 (62, 70)	>0.9
Type 2 diabetes mellitus (n(%))	50 (48%)	66 (57%)	0.15	32 (43%)	30 (41%)	0.8
Any comorbidity	90 (86%)	104 (90%)	0.3	71 (95%)	64 (86%)	0.087
Antibiotic intake	0 (0%)	2 (1.9%)	0.5	0 (0%)	4 (6.1%)	0.051
Drugs use	24 (23%)	29 (25%)	0.7	13 (17%)	9 (12%)	0.4
Smoke			0.2			0.8
Current	4 (3.8%)	4 (3.5%)		3 (4.0%)	5 (6.8%)	
Quit	6 (5.7%)	15 (13%)		9 (12%)	9 (12%)	
Never	95 (90%)	96 (83%)		63 (84%)	60 (81%)	
Alcohol			0.13			0.11
Current	7 (6.7%)	16 (14%)		9 (12%)	15 (20%)	
Quit	3 (2.9%)	6 (5.2%)		7 (9.3%)	2 (2.7%)	
Never	95 (90%)	93 (81%)		59 (79%)	57 (77%)	

[1]Categorical data are presented as number (percentage) and continuous data as median (IQR)

[2]Pearson's Chi-squared test; Wilcoxon rank sum test; Fisher's exact test

Table S2. The fold change of SIM01 species (BABBBL_fc) between subjects' baseline gut microbiome with or without specific KEGG Orthology (KO).

Baseline KO	Median BABBBL_fc of KO		Outcome	Prevlance	*p* value	FDR	Pathway Level 1	Pathway
	absent group	present group						
K02671	7.6913	0.7959	BABBBL_fc	0.4082	0.0000	0.0017	Brite Hierarchies	Secretion system [BR:ko02044]
K07768	8.1736	1.0822	BABBBL_fc	0.5102	0.0000	0.0017	Environmental Information Processing	Two-component system [PATH:ko02020]
K08168	10.3060	1.6205	BABBBL_fc	0.7279	0.0000	0.0065	Brite Hierarchies	Transporters [BR:ko02000]
K15531	14.4288	1.9190	BABBBL_fc	0.9048	0.0000	0.0065	Not Included in Pathway or Brite	Enzymes with EC numbers
K22306	7.7266	0.9722	BABBBL_fc	0.4286	0.0000	0.0065	Not Included in Pathway or Brite	Enzymes with EC numbers
K02077	8.3061	1.6276	BABBBL_fc	0.6803	0.0001	0.0065	Brite Hierarchies	Transporters [BR:ko02000]
K16148	8.2615	1.6163	BABBBL_fc	0.6327	0.0000	0.0065	Metabolism	Starch and sucrose metabolism [PATH:ko00500]
K07457	6.4935	0.1932	BABBBL_fc	0.2109	0.0001	0.0065	Not Included in Pathway or Brite	Replication and repair
K16925	8.3061	1.6184	BABBBL_fc	0.7211	0.0001	0.0101	Brite Hierarchies	Transporters [BR:ko02000]
K04092	8.2170	1.6297	BABBBL_fc	0.7347	0.0001	0.0123	Metabolism	Phenylalanine, tyrosine and tryptophan biosynthesis [PATH:ko00400]
K07177	8.2360	1.6276	BABBBL_fc	0.6939	0.0003	0.0204	Brite Hierarchies	Peptidases and inhibitors [BR:ko01002]
K01697	8.2170	1.7295	BABBBL_fc	0.7211	0.0005	0.0261	Metabolism	Glycine, serine and threonine metabolism [PATH:ko00260]
K01779	7.0938	0.7363	BABBBL_fc	0.4014	0.0004	0.0261	Metabolism	Alanine, aspartate and glutamate metabolism [PATH:ko00250]
K01858	7.9624	1.6389	BABBBL_fc	0.6871	0.0005	0.0261	Metabolism	Inositol phosphate metabolism [PATH:ko00562]
K17195	9.2586	1.8423	BABBBL_fc	0.8299	0.0005	0.0261	Metabolism	Fructose and mannose metabolism [PATH:ko00051]
K01227	8.2045	1.6389	BABBBL_fc	0.6599	0.0005	0.0261	Metabolism	Other glycan degradation [PATH:ko00511]
K13940	10.8727	1.8917	BABBBL_fc	0.8844	0.0004	0.0261	Metabolism	Folate biosynthesis [PATH:ko00790]
K06330	8.2615	1.6389	BABBBL_fc	0.8095	0.0005	0.0261	Not Included in Pathway or Brite	Cell growth
K07260	11.1979	1.9823	BABBBL_fc	0.9116	0.0007	0.0302	Metabolism	Peptidoglycan biosynthesis [PATH:ko00550]
K07503	8.1919	1.6889	BABBBL_fc	0.7483	0.0007	0.0319	Not Included in Pathway or Brite	Enzymes with EC numbers
K16926	12.6902	2.0750	BABBBL_fc	0.9320	0.0009	0.0341	Brite Hierarchies	Transporters [BR:ko02000]

K21012	7.9932	1.5503	BABBBL_fc	0.5782	0.0008	0.0341	Cellular Processes	Biofilm formation - Pseudomonas aeruginosa [PATH:ko02025]
K07533	16.2377	2.9273	BABBBL_fc	0.9524	0.0010	0.0388	Brite Hierarchies	Chaperones and folding catalysts [BR:ko03110]
K07386	10.8727	1.9823	BABBBL_fc	0.9252	0.0012	0.0444	Brite Hierarchies	Peptidases and inhibitors [BR:ko01002]
K04751	16.4351	4.0810	BABBBL_fc	0.9660	0.0015	0.0486	Environmental Information Processing	Two-component system [PATH:ko02020]
K13527	8.1832	1.7388	BABBBL_fc	0.7007	0.0015	0.0486	Genetic Information Processing	Proteasome [PATH:ko03050]

Table S3. Correlations between the fold change of the relative abundance of SIM01-contained three species (BABBBL_fc) and sVNT levels.

Treatment	Vaccine type	M6 vaccine dose	Immune Outcome	Var	*p* value	Spearman's Rho
SIM01	BNT162b2	-	IgG levels at 1-month postvaccination	BABBBL_fc	**0.0188**	0.2745
Placebo	BNT162b2	-	IgG levels at 1-month postvaccination	BABBBL_fc	0.7844	0.0361
SIM01	CoronaVac	-	IgG levels at 1-month postvaccination	BABBBL_fc	0.0680	-0.3384
Placebo	CoronaVac	-	IgG levels at 1-month postvaccination	BABBBL_fc	0.2365	0.3909
Placebo	BNT162b2	-	sVNT levels at 1-month postvaccination	BABBBL_fc	0.1252	0.1707
SIM01	BNT162b2	-	sVNT levels at 1-month postvaccination	BABBBL_fc	0.0550	0.2256
Placebo	CoronaVac	-	sVNT levels at 1-month postvaccination	BABBBL_fc	0.1485	0.2532
SIM01	CoronaVac	-	sVNT levels at 1-month postvaccination	BABBBL_fc	0.2521	-0.2192
Placebo	BNT162b2	2nd dose	sVNT levels at 6-month postvaccination	BABBBL_fc	0.3596	0.1146
SIM01	BNT162b2	2nd dose	sVNT levels at 6-month postvaccination	BABBBL_fc	**0.0374**	0.2409
Placebo	CoronaVac	2nd dose	sVNT levels at 6-month postvaccination	BABBBL_fc	0.4444	0.2736
SIM01	CoronaVac	2nd dose	sVNT levels at 6-month postvaccination	BABBBL_fc	0.7162	-0.1189
Placebo	BNT162b2	3rd dose	sVNT levels at 6-month postvaccination	BABBBL_fc	0.5898	0.1283
SIM01	BNT162b2	3rd dose	sVNT levels at 6-month postvaccination	BABBBL_fc	**0.0432**	0.4159
Placebo	CoronaVac	3rd dose	sVNT levels at 6-month postvaccination	BABBBL_fc	0.2157	0.2819
SIM01	CoronaVac	3rd dose	sVNT levels at 6-month postvaccination	BABBBL_fc	0.8438	0.0593

postvaccination, post 2nd vaccination dose

Table S4. Differential gut metabolites between baseline and 1-month postvaccination within SIM01 arm after adjusting vaccine type.

feature	metadata	value	coef	stderr	N	N.not.0	pval	qval
L-Sorbose	timepoint	M1	1.5692	0.5722	288	288	0.0069	0.1287
Glyceraldehyde	timepoint	M1	1.5024	0.4006	288	288	0.0003	0.0239
Ricinoleic acid	timepoint	M1	1.3829	0.4885	288	288	0.0050	0.1210
Taurocyamine	timepoint	M1	1.2694	0.4122	288	288	0.0025	0.0847
Histamine	timepoint	M1	1.2402	0.4520	288	288	0.0068	0.1287
N-Formylmethionine	timepoint	M1	1.2124	0.4315	288	288	0.0053	0.1210
3-Mercaptolactic acid	timepoint	M1	1.1510	0.3636	288	288	0.0019	0.0732
3-Aminobutanoic acid	timepoint	M1	1.1332	0.3999	288	288	0.0053	0.1210
2-Aminoisobutyric acid	timepoint	M1	1.1159	0.3464	288	288	0.0016	0.0698
Mannitol	timepoint	M1	0.8768	0.3387	288	288	0.0106	0.1668
Ferulic acid	timepoint	M1	0.8646	0.3610	288	288	0.0179	0.2390
myo-Inositol	timepoint	M1	0.8334	0.3271	288	288	0.0119	0.1724
Phthalic acid	timepoint	M1	0.7680	0.2631	288	288	0.0041	0.1127
3-Hydroxyvaleric acid	timepoint	M1	0.7526	0.2898	288	288	0.0104	0.1668
2-hydroxyadipic acid	timepoint	M1	0.6693	0.2654	288	288	0.0128	0.1808
Imidazoleacetic acid	timepoint	M1	0.6604	0.2388	288	288	0.0064	0.1287
2-Acetylpyrazine	timepoint	M1	0.6599	0.2417	288	288	0.0071	0.1292
Vanillic acid	timepoint	M1	0.6541	0.2211	288	288	0.0036	0.1050
Benzoic acid	timepoint	M1	0.6310	0.2474	288	288	0.0118	0.1724
Ascorbic acid	timepoint	M1	0.5716	0.1785	288	288	0.0017	0.0698
Lactulose	timepoint	M1	0.5014	0.1848	288	288	0.0075	0.1317
Senecioic acid	timepoint	M1	0.4091	0.1476	288	288	0.0063	0.1287
Pyridoxal	timepoint	M1	0.2517	0.0947	288	288	0.0087	0.1489
N2-Acetylornithine	timepoint	M1	-0.5897	0.2054	288	288	0.0047	0.1210
Thiodiacetic acid	Vaccine.Type	Sinovac	2.5714	0.7035	288	288	0.0004	0.0239
4-Methylhexanoic acid	Vaccine.Type	Sinovac	1.6547	0.6421	288	288	0.0110	0.1677

N-Acetylasparagine	Vaccine.Type	Sinovac	1.6162	0.4906	288	288	0.0012	0.0602
Xanthosine	Vaccine.Type	Sinovac	1.5267	0.6429	288	288	0.0189	0.2437
Sinapic acid	Vaccine.Type	Sinovac	0.9486	0.3453	288	288	0.0068	0.1287
L-Gulonolactone	Vaccine.Type	Sinovac	0.3932	0.1403	288	288	0.0054	0.1210
Etiadienic Acid Methyl Ester	Vaccine.Type	Sinovac	0.2992	0.0811	288	288	0.0003	0.0239
5-Hydroxyindoleacetic acid	Vaccine.Type	Sinovac	-0.3546	0.1483	288	288	0.0181	0.2390
N-Acetylleucine	Vaccine.Type	Sinovac	-0.5161	0.1710	288	288	0.0030	0.0918
N2-Acetylornithine	Vaccine.Type	Sinovac	-0.9064	0.2162	288	288	0.0000	0.0094
6-Hydroxynicotinic acid	Vaccine.Type	Sinovac	-1.0557	0.4020	288	288	0.0096	0.1587
Meta-Tyrosine	Vaccine.Type	Sinovac	-1.1641	0.3805	288	288	0.0024	0.0847
Alloisolithocholic Acid(AILCA)	Vaccine.Type	Sinovac	-1.2737	0.3492	288	288	0.0004	0.0239
Epicatechin	Vaccine.Type	Sinovac	-1.4233	0.5841	288	288	0.0161	0.2217
Glycolic acid	Vaccine.Type	Sinovac	-1.4739	0.2749	288	288	0.0000	0.0001
N-alpha-Acetyl-L-lysine	Vaccine.Type	Sinovac	-1.5429	0.3185	288	288	0.0000	0.0006
Trigonelline	Vaccine.Type	Sinovac	-1.6828	0.4766	288	288	0.0006	0.0324
Ricinoleic acid	Vaccine.Type	Sinovac	-1.9792	0.4932	288	288	0.0001	0.0111
3-Pyridylacetic acid	Vaccine.Type	Sinovac	-2.1733	0.6579	288	288	0.0012	0.0602
Methylimidazoleacetic acid	Vaccine.Type	Sinovac	-2.5013	0.6692	288	288	0.0003	0.0239
Imidazolepropionic acid	Vaccine.Type	Sinovac	-2.8084	0.9171	288	288	0.0026	0.0847

Table S5. Differential gut metabolites between baseline and 1-month postvaccination within placebo arm after adjusting vaccine type.

feature	metadata	value	coef	stderr	N	N.not.0	*p* value	qval
Xanthosine	timepoint	M1	1.6896	0.5066	264.0	264.0	0.0011	0.0402
Thiodiacetic acid	timepoint	M1	1.6696	0.6391	264.0	264.0	0.0095	0.1402
Inosine	timepoint	M1	1.4480	0.6055	264.0	264.0	0.0182	0.1773
p-Hydroxyphenylacetic acid	timepoint	M1	1.3764	0.5696	264.0	264.0	0.0170	0.1773
Histamine	timepoint	M1	1.2501	0.4963	264.0	264.0	0.0130	0.1536
Ursocholic Acid(UCA)	timepoint	M1	1.2402	0.5363	264.0	264.0	0.0223	0.1960
2-Methylpentanoic acid	timepoint	M1	1.1398	0.3665	264.0	264.0	0.0023	0.0618
3-Methoxybenzenepropanoic acid	timepoint	M1	1.1336	0.4590	264.0	264.0	0.0148	0.1686
N-Acetylasparagine	timepoint	M1	1.0911	0.3556	264.0	264.0	0.0026	0.0618
Orotic acid	timepoint	M1	1.0589	0.4544	264.0	264.0	0.0213	0.1944
Glyceraldehyde	timepoint	M1	1.0148	0.4530	264.0	264.0	0.0259	0.2089
Syringic acid	timepoint	M1	1.0019	0.3605	264.0	264.0	0.0063	0.1019
2-hydroxyadipic acid	timepoint	M1	1.0014	0.3609	264.0	264.0	0.0063	0.1019
3-Hydroxyadipic acid	timepoint	M1	0.9550	0.3691	264.0	264.0	0.0108	0.1402
L-Methionine(Met)	timepoint	M1	0.7872	0.3264	264.0	264.0	0.0173	0.1773
Vanillic acid	timepoint	M1	0.7059	0.3043	264.0	264.0	0.0219	0.1955
Dodecanedioic acid	timepoint	M1	0.6875	0.3137	264.0	264.0	0.0302	0.2190
Imidazoleacetic acid	timepoint	M1	0.6596	0.2948	264.0	264.0	0.0270	0.2124
Senecioic acid	timepoint	M1	0.6234	0.2876	264.0	264.0	0.0320	0.2262
Acetylglycine	timepoint	M1	0.5553	0.2598	264.0	264.0	0.0344	0.2348
O-Acetylserine	timepoint	M1	0.5298	0.2042	264.0	264.0	0.0105	0.1402
12-Ketodeoxycholic acid(12-keto-CDCA)	timepoint	M1	0.5148	0.2270	264.0	264.0	0.0250	0.2041
L-Serine(Ser)	timepoint	M1	0.5054	0.2358	264.0	264.0	0.0339	0.2343
23-Norcholic Acid	timepoint	M1	0.5038	0.2282	264.0	264.0	0.0290	0.2158
N-Acetylserine	timepoint	M1	0.3062	0.1418	264.0	264.0	0.0326	0.2277
Bicine	timepoint	M1	0.2893	0.1269	264.0	264.0	0.0242	0.2034

Galactosamine	timepoint	M1	0.2483	0.1032	264.0	264.0	0.0175	0.1773
Tiglylglycine	timepoint	M1	0.1642	0.0583	264.0	264.0	0.0056	0.0987
Acetic acid	timepoint	M1	-0.1417	0.0604	264.0	264.0	0.0205	0.1919
3-Methylpentanoic acid	Vaccine.Type	Sinovac	2.7814	0.6189	264.0	264.0	<0.0001	0.0022
Thiodiacetic acid	Vaccine.Type	Sinovac	2.6338	0.6482	264.0	264.0	<0.0001	0.0062
Glycodeoxycholic acid(GDCA)	Vaccine.Type	Sinovac	2.1607	0.4971	264.0	264.0	<0.0001	0.0032
4-Methylhexanoic acid	Vaccine.Type	Sinovac	2.1432	0.6775	264.0	264.0	0.0019	0.0593
N-Acetylasparagine	Vaccine.Type	Sinovac	2.1324	0.5436	264.0	264.0	0.0001	0.0102
4-Hydroxybenzaldehyde	Vaccine.Type	Sinovac	2.0055	0.5732	264.0	264.0	0.0006	0.0286
N-Methyl-D-aspartic acid	Vaccine.Type	Sinovac	1.8973	0.6193	264.0	264.0	0.0027	0.0618
Oxindole-3-acetic acid	Vaccine.Type	Sinovac	1.7350	0.6142	264.0	264.0	0.0055	0.0987
3-Methoxybenzenepropanoic acid	Vaccine.Type	Sinovac	1.6926	0.5138	264.0	264.0	0.0013	0.0434
2-Methylpentanoic acid	Vaccine.Type	Sinovac	1.5600	0.4148	264.0	264.0	0.0003	0.0148
3-Methyladipic acid	Vaccine.Type	Sinovac	1.5320	0.4953	264.0	264.0	0.0024	0.0618
Orotic acid	Vaccine.Type	Sinovac	1.2628	0.5245	264.0	264.0	0.0175	0.1773
3-Hydroxydodecanedioic acid	Vaccine.Type	Sinovac	1.2585	0.5159	264.0	264.0	0.0161	0.1758
Apocholic Acid(APCA)	Vaccine.Type	Sinovac	1.2063	0.4600	264.0	264.0	0.0098	0.1402
Dodecanedioic acid	Vaccine.Type	Sinovac	1.1673	0.3895	264.0	264.0	0.0033	0.0701
Methylglutaric acid	Vaccine.Type	Sinovac	1.1264	0.4705	264.0	264.0	0.0181	0.1773
Tetradecenoic acid	Vaccine.Type	Sinovac	1.0759	0.4081	264.0	264.0	0.0094	0.1402
Caprylic acid	Vaccine.Type	Sinovac	1.0423	0.4768	264.0	264.0	0.0306	0.2191
12-Dehydrocholic Acid Diacetate	Vaccine.Type	Sinovac	0.9838	0.3433	264.0	264.0	0.0049	0.0908
7-Ketolithocholic acid(7-keto-LCA)	Vaccine.Type	Sinovac	0.9260	0.3195	264.0	264.0	0.0044	0.0863
Suberic acid	Vaccine.Type	Sinovac	0.8915	0.2998	264.0	264.0	0.0035	0.0727
Myristoleic acid	Vaccine.Type	Sinovac	0.8670	0.3957	264.0	264.0	0.0302	0.2190
Azelaic acid	Vaccine.Type	Sinovac	0.8264	0.3644	264.0	264.0	0.0250	0.2041
Oxalacetic acid	Vaccine.Type	Sinovac	0.7354	0.2961	264.0	264.0	0.0143	0.1658
Benzoic acid	Vaccine.Type	Sinovac	0.7089	0.2975	264.0	264.0	0.0186	0.1773

Choleic Acid	Vaccine.Type	Sinovac	0.7001	0.3145	264.0	264.0	0.0277	0.2124
Senecioic acid	Vaccine.Type	Sinovac	0.6864	0.3076	264.0	264.0	0.0274	0.2124
Isovalerylglycine	Vaccine.Type	Sinovac	0.6366	0.2275	264.0	264.0	0.0059	0.1008
3-Hydroxysebacic acid	Vaccine.Type	Sinovac	0.6320	0.2715	264.0	264.0	0.0215	0.1944
Menadione(Vitamin K3)	Vaccine.Type	Sinovac	0.6222	0.2607	264.0	264.0	0.0184	0.1773
Indoleacrylic acid	Vaccine.Type	Sinovac	0.5910	0.2672	264.0	264.0	0.0278	0.2124
Pyridoxal 5'-phosphate	Vaccine.Type	Sinovac	0.5171	0.1694	264.0	264.0	0.0028	0.0618
Monomethyl glutaric acid	Vaccine.Type	Sinovac	0.3612	0.1402	264.0	264.0	0.0111	0.1402
Etiadienic Acid Methyl Ester	Vaccine.Type	Sinovac	0.3246	0.0885	264.0	264.0	0.0004	0.0178
2-Ketobutyric acid	Vaccine.Type	Sinovac	0.2975	0.0731	264.0	264.0	<0.0001	0.0067
Glucose	Vaccine.Type	Sinovac	0.0837	0.0377	264.0	264.0	0.0283	0.2133
N-Acetylleucine	Vaccine.Type	Sinovac	-0.7696	0.3128	264.0	264.0	0.0152	0.1693
Lactulose	Vaccine.Type	Sinovac	-0.8686	0.3427	264.0	264.0	0.0124	0.1504
Glycyl-glycine	Vaccine.Type	Sinovac	-0.9632	0.4177	264.0	264.0	0.0227	0.1965
Alanylphenylalanine	Vaccine.Type	Sinovac	-1.0025	0.3929	264.0	264.0	0.0113	0.1402
Meta-Tyrosine	Vaccine.Type	Sinovac	-1.0835	0.4250	264.0	264.0	0.0114	0.1402
Mannose	Vaccine.Type	Sinovac	-1.0921	0.4756	264.0	264.0	0.0233	0.1984
3-Hydroxyadipic acid	Vaccine.Type	Sinovac	-1.1243	0.4355	264.0	264.0	0.0110	0.1402
2-hydroxyadipic acid	Vaccine.Type	Sinovac	-1.1368	0.4339	264.0	264.0	0.0098	0.1402
Cystine	Vaccine.Type	Sinovac	-1.2014	0.4189	264.0	264.0	0.0045	0.0863
N2-Acetylornithine	Vaccine.Type	Sinovac	-1.2944	0.3539	264.0	264.0	0.0004	0.0178
6-Hydroxynicotinic acid	Vaccine.Type	Sinovac	-1.3174	0.3874	264.0	264.0	0.0009	0.0370
Phenylglyoxylic acid	Vaccine.Type	Sinovac	-1.4208	0.4584	264.0	264.0	0.0024	0.0618
Ricinoleic acid	Vaccine.Type	Sinovac	-1.4575	0.5633	264.0	264.0	0.0108	0.1402
Alloisolithocholic Acid(AILCA)	Vaccine.Type	Sinovac	-1.5568	0.4012	264.0	264.0	0.0002	0.0106
Trigonelline	Vaccine.Type	Sinovac	-1.8922	0.5908	264.0	264.0	0.0017	0.0552
Methylimidazoleacetic acid	Vaccine.Type	Sinovac	-1.9359	0.6346	264.0	264.0	0.0028	0.0618
Glycolic acid	Vaccine.Type	Sinovac	-2.2094	0.3824	264.0	264.0	<0.0001	<0.0001

N-alpha-Acetyl-L-lysine	Vaccine.Type	Sinovac	-2.3954	0.4025	264.0	264.0	<0.0001	<0.0001
Imidazolepropionic acid	Vaccine.Type	Sinovac	-3.2454	0.9656	264.0	264.0	0.0010	0.0395
3-Pyridylacetic acid	Vaccine.Type	Sinovac	-3.5953	0.7953	264.0	264.0	<0.0001	0.0022

Table S6. Correlations between the fold change of the relative abundance of SIM01-contained three species (BABBBL_fc) and the fold change of differential metabolites between baseline and 1-month postvaccination.

treatment	VACCINE_type	Variable1	Variable2	Correlation	*p* value
**SIM01**	**BioNTech**	**N-Formylmethionine**	**s__Bifidobacterium_bifidum_fc**	**0.3261**	**0.0040**
**SIM01**	**BioNTech**	**Benzoic acid**	**s__Bifidobacterium_bifidum_fc**	**0.2860**	**0.0123**
**SIM01**	**BioNTech**	**myo-Inositol**	**s__Bifidobacterium_bifidum_fc**	**0.2478**	**0.0309**
SIM01	BioNTech	L-Sorbose	s__Bifidobacterium_bifidum_fc	0.2244	0.0514
SIM01	BioNTech	Ricinoleic acid	BABBBL_fc	0.2243	0.0514
SIM01	BioNTech	3-Hydroxyvaleric acid	s__Bifidobacterium_adolescentis_fc	0.2101	0.0685
SIM01	BioNTech	2-Acetylpyrazine	s__Bifidobacterium_longum_fc	0.2071	0.0726
SIM01	BioNTech	Benzoic acid	BABBBL_fc	0.2067	0.0733
SIM01	BioNTech	Taurocyamine	s__Bifidobacterium_adolescentis_fc	-0.2055	0.0750
SIM01	BioNTech	N-Formylmethionine	s__Bifidobacterium_adolescentis_fc	0.2020	0.0801
SIM01	BioNTech	Ricinoleic acid	s__Bifidobacterium_adolescentis_fc	0.1990	0.0848
SIM01	BioNTech	Mannitol	s__Bifidobacterium_bifidum_fc	0.1984	0.0858
SIM01	BioNTech	N-Formylmethionine	BABBBL_fc	0.1955	0.0905
SIM01	BioNTech	3-Hydroxyvaleric acid	s__Bifidobacterium_bifidum_fc	0.1943	0.0926
SIM01	BioNTech	Ferulic acid	s__Bifidobacterium_bifidum_fc	0.1851	0.1094
SIM01	BioNTech	3-Hydroxyvaleric acid	BABBBL_fc	0.1831	0.1134
SIM01	BioNTech	Benzoic acid	s__Bifidobacterium_longum_fc	0.1821	0.1154
SIM01	BioNTech	Ferulic acid	s__Bifidobacterium_longum_fc	-0.1749	0.1309
SIM01	BioNTech	Phthalic acid	s__Bifidobacterium_adolescentis_fc	-0.1630	0.1594
SIM01	BioNTech	Pyridoxal	s__Bifidobacterium_bifidum_fc	0.1602	0.1668
SIM01	BioNTech	3-Mercaptolactic acid	s__Bifidobacterium_longum_fc	0.1513	0.1919
SIM01	BioNTech	Phthalic acid	BABBBL_fc	-0.1463	0.2074
SIM01	BioNTech	Ricinoleic acid	s__Bifidobacterium_longum_fc	0.1428	0.2186
SIM01	BioNTech	Lactulose	s__Bifidobacterium_bifidum_fc	0.1369	0.2381
SIM01	BioNTech	N2-Acetylornithine	BABBBL_fc	-0.1342	0.2479

SIM01	BioNTech	Pyridoxal	BABBBL_fc	0.1317	0.2569
SIM01	BioNTech	Taurocyamine	BABBBL_fc	-0.1305	0.2611
SIM01	BioNTech	N2-Acetylornithine	s__Bifidobacterium_longum_fc	-0.1272	0.2737
SIM01	BioNTech	2-Acetylpyrazine	s__Bifidobacterium_bifidum_fc	0.1262	0.2772
SIM01	BioNTech	2-Aminoisobutyric acid	s__Bifidobacterium_adolescentis_fc	0.1201	0.3014
SIM01	BioNTech	Pyridoxal	s__Bifidobacterium_adolescentis_fc	0.1193	0.3047
SIM01	BioNTech	3-Aminobutanoic acid	s__Bifidobacterium_adolescentis_fc	0.1187	0.3071
SIM01	BioNTech	3-Hydroxyvaleric acid	s__Bifidobacterium_longum_fc	0.1181	0.3096
SIM01	BioNTech	N2-Acetylornithine	s__Bifidobacterium_adolescentis_fc	-0.1173	0.3128
SIM01	BioNTech	2-Acetylpyrazine	BABBBL_fc	0.1129	0.3315
SIM01	BioNTech	Phthalic acid	s__Bifidobacterium_bifidum_fc	0.1120	0.3356
SIM01	BioNTech	Phthalic acid	s__Bifidobacterium_longum_fc	-0.1081	0.3527
SIM01	BioNTech	3-Mercaptolactic acid	s__Bifidobacterium_bifidum_fc	0.1073	0.3564
SIM01	BioNTech	Benzoic acid	s__Bifidobacterium_adolescentis_fc	0.1068	0.3584
SIM01	BioNTech	Lactulose	s__Bifidobacterium_adolescentis_fc	-0.1050	0.3667
SIM01	BioNTech	3-Aminobutanoic acid	BABBBL_fc	0.1049	0.3672
SIM01	BioNTech	3-Aminobutanoic acid	s__Bifidobacterium_bifidum_fc	0.1044	0.3696
SIM01	BioNTech	Ricinoleic acid	s__Bifidobacterium_bifidum_fc	0.1019	0.3809
SIM01	BioNTech	Ascorbic acid	s__Bifidobacterium_bifidum_fc	0.1017	0.3820
SIM01	BioNTech	2-Aminoisobutyric acid	BABBBL_fc	0.0971	0.4042
SIM01	BioNTech	Mannitol	s__Bifidobacterium_longum_fc	-0.0959	0.4100
SIM01	BioNTech	3-Mercaptolactic acid	s__Bifidobacterium_adolescentis_fc	0.0952	0.4133
SIM01	BioNTech	2-Aminoisobutyric acid	s__Bifidobacterium_bifidum_fc	0.0914	0.4326
SIM01	BioNTech	Lactulose	s__Bifidobacterium_longum_fc	-0.0812	0.4855
SIM01	BioNTech	3-Mercaptolactic acid	BABBBL_fc	0.0787	0.4990
SIM01	BioNTech	myo-Inositol	s__Bifidobacterium_longum_fc	-0.0766	0.5105
SIM01	BioNTech	Mannitol	s__Bifidobacterium_adolescentis_fc	0.0733	0.5290
SIM01	BioNTech	2-Acetylpyrazine	s__Bifidobacterium_adolescentis_fc	0.0725	0.5337

SIM01	BioNTech	Lactulose	BABBBL_fc	-0.0709	0.5431
SIM01	BioNTech	L-Sorbose	s__Bifidobacterium_adolescentis_fc	-0.0588	0.6139
SIM01	BioNTech	Ascorbic acid	BABBBL_fc	0.0560	0.6307
SIM01	BioNTech	Taurocyamine	s__Bifidobacterium_bifidum_fc	0.0479	0.6814
SIM01	BioNTech	N2-Acetylornithine	s__Bifidobacterium_bifidum_fc	-0.0465	0.6901
SIM01	BioNTech	myo-Inositol	BABBBL_fc	-0.0436	0.7083
SIM01	BioNTech	Mannitol	BABBBL_fc	0.0432	0.7111
SIM01	BioNTech	Ascorbic acid	s__Bifidobacterium_adolescentis_fc	0.0397	0.7333
SIM01	BioNTech	myo-Inositol	s__Bifidobacterium_adolescentis_fc	-0.0371	0.7500
SIM01	BioNTech	L-Sorbose	BABBBL_fc	-0.0362	0.7560
SIM01	BioNTech	Pyridoxal	s__Bifidobacterium_longum_fc	0.0345	0.7673
SIM01	BioNTech	L-Sorbose	s__Bifidobacterium_longum_fc	-0.0306	0.7929
SIM01	BioNTech	Ascorbic acid	s__Bifidobacterium_longum_fc	-0.0299	0.7976
SIM01	BioNTech	Ferulic acid	s__Bifidobacterium_adolescentis_fc	0.0234	0.8409
SIM01	BioNTech	Taurocyamine	s__Bifidobacterium_longum_fc	-0.0215	0.8539
SIM01	BioNTech	N-Formylmethionine	s__Bifidobacterium_longum_fc	-0.0145	0.9012
SIM01	BioNTech	Ferulic acid	BABBBL_fc	-0.0065	0.9559
SIM01	BioNTech	2-Aminoisobutyric acid	s__Bifidobacterium_longum_fc	0.0044	0.9697
SIM01	BioNTech	3-Aminobutanoic acid	s__Bifidobacterium_longum_fc	0.0021	0.9856
**SIM01**	**Sinovac**	**Pyridoxal**	**s__Bifidobacterium_bifidum_fc**	**-0.5712**	**0.0044**
**SIM01**	**Sinovac**	**Taurocyamine**	**s__Bifidobacterium_longum_fc**	**0.4980**	**0.0167**
**SIM01**	**Sinovac**	**myo-Inositol**	**s__Bifidobacterium_adolescentis_fc**	**-0.4522**	**0.0303**
**SIM01**	**Sinovac**	**L-Sorbose**	**s__Bifidobacterium_adolescentis_fc**	**-0.4463**	**0.0328**
**SIM01**	**Sinovac**	**Ricinoleic acid**	**s__Bifidobacterium_adolescentis_fc**	**-0.4378**	**0.0367**
SIM01	Sinovac	Ferulic acid	BABBBL_fc	-0.3715	0.0817
SIM01	Sinovac	Lactulose	s__Bifidobacterium_adolescentis_fc	-0.3596	0.0919
SIM01	Sinovac	Pyridoxal	BABBBL_fc	-0.3587	0.0934
SIM01	Sinovac	Taurocyamine	s__Bifidobacterium_bifidum_fc	0.3470	0.1048

SIM01	Sinovac	L-Sorbose	BABBBL_fc	-0.3330	0.1208
SIM01	Sinovac	Taurocyamine	BABBBL_fc	0.3320	0.1219
SIM01	Sinovac	Phthalic acid	s__Bifidobacterium_bifidum_fc	-0.3119	0.1474
SIM01	Sinovac	Mannitol	BABBBL_fc	-0.3053	0.1563
SIM01	Sinovac	myo-Inositol	BABBBL_fc	-0.2994	0.1649
SIM01	Sinovac	Phthalic acid	BABBBL_fc	-0.2984	0.1663
SIM01	Sinovac	Benzoic acid	s__Bifidobacterium_longum_fc	0.2945	0.1722
SIM01	Sinovac	N2-Acetylornithine	s__Bifidobacterium_adolescentis_fc	-0.2947	0.1722
SIM01	Sinovac	Ascorbic acid	s__Bifidobacterium_adolescentis_fc	-0.2883	0.1822
SIM01	Sinovac	myo-Inositol	s__Bifidobacterium_bifidum_fc	-0.2856	0.1865
SIM01	Sinovac	2-Aminoisobutyric acid	BABBBL_fc	-0.2796	0.1956
SIM01	Sinovac	3-Hydroxyvaleric acid	BABBBL_fc	-0.2777	0.1989
SIM01	Sinovac	3-Aminobutanoic acid	BABBBL_fc	-0.2638	0.2229
SIM01	Sinovac	Lactulose	s__Bifidobacterium_bifidum_fc	-0.2593	0.2322
SIM01	Sinovac	Lactulose	BABBBL_fc	-0.2579	0.2338
SIM01	Sinovac	Mannitol	s__Bifidobacterium_adolescentis_fc	-0.2571	0.2364
SIM01	Sinovac	Phthalic acid	s__Bifidobacterium_adolescentis_fc	-0.2526	0.2449
SIM01	Sinovac	Ascorbic acid	s__Bifidobacterium_longum_fc	0.2480	0.2527
SIM01	Sinovac	L-Sorbose	s__Bifidobacterium_bifidum_fc	-0.2212	0.3105
SIM01	Sinovac	2-Aminoisobutyric acid	s__Bifidobacterium_bifidum_fc	-0.2206	0.3117
SIM01	Sinovac	Lactulose	s__Bifidobacterium_longum_fc	0.2164	0.3198
SIM01	Sinovac	Ferulic acid	s__Bifidobacterium_adolescentis_fc	-0.2125	0.3304
SIM01	Sinovac	N-Formylmethionine	s__Bifidobacterium_adolescentis_fc	-0.2110	0.3338
SIM01	Sinovac	N-Formylmethionine	BABBBL_fc	-0.2006	0.3571
SIM01	Sinovac	L-Sorbose	s__Bifidobacterium_longum_fc	0.1818	0.4046
SIM01	Sinovac	myo-Inositol	s__Bifidobacterium_longum_fc	0.1779	0.4151
SIM01	Sinovac	Ascorbic acid	BABBBL_fc	-0.1640	0.4528
SIM01	Sinovac	Benzoic acid	BABBBL_fc	0.1462	0.5039

SIM01	Sinovac	Mannitol	s__Bifidobacterium_bifidum_fc	-0.1449	0.5096
SIM01	Sinovac	2-Acetylpyrazine	BABBBL_fc	-0.1334	0.5424
SIM01	Sinovac	N-Formylmethionine	s__Bifidobacterium_longum_fc	0.1255	0.5669
SIM01	Sinovac	Benzoic acid	s__Bifidobacterium_bifidum_fc	-0.1242	0.5722
SIM01	Sinovac	N-Formylmethionine	s__Bifidobacterium_bifidum_fc	-0.1139	0.6047
SIM01	Sinovac	Benzoic acid	s__Bifidobacterium_adolescentis_fc	0.1139	0.6048
SIM01	Sinovac	3-Hydroxyvaleric acid	s__Bifidobacterium_adolescentis_fc	-0.1134	0.6064
SIM01	Sinovac	3-Mercaptolactic acid	BABBBL_fc	-0.1126	0.6076
SIM01	Sinovac	N2-Acetylornithine	s__Bifidobacterium_bifidum_fc	0.1093	0.6196
SIM01	Sinovac	Pyridoxal	s__Bifidobacterium_adolescentis_fc	-0.1065	0.6287
SIM01	Sinovac	2-Acetylpyrazine	s__Bifidobacterium_adolescentis_fc	-0.1030	0.6399
SIM01	Sinovac	3-Aminobutanoic acid	s__Bifidobacterium_bifidum_fc	-0.0938	0.6702
SIM01	Sinovac	Taurocyamine	s__Bifidobacterium_adolescentis_fc	-0.0921	0.6759
SIM01	Sinovac	Ascorbic acid	s__Bifidobacterium_bifidum_fc	-0.0809	0.7135
SIM01	Sinovac	Ferulic acid	s__Bifidobacterium_longum_fc	0.0761	0.7296
SIM01	Sinovac	Ricinoleic acid	BABBBL_fc	-0.0672	0.7604
SIM01	Sinovac	3-Hydroxyvaleric acid	s__Bifidobacterium_longum_fc	-0.0652	0.7673
SIM01	Sinovac	3-Mercaptolactic acid	s__Bifidobacterium_adolescentis_fc	-0.0634	0.7738
SIM01	Sinovac	2-Aminoisobutyric acid	s__Bifidobacterium_longum_fc	0.0613	0.7812
SIM01	Sinovac	3-Mercaptolactic acid	s__Bifidobacterium_bifidum_fc	0.0603	0.7846
SIM01	Sinovac	3-Aminobutanoic acid	s__Bifidobacterium_longum_fc	0.0603	0.7846
SIM01	Sinovac	2-Acetylpyrazine	s__Bifidobacterium_longum_fc	0.0573	0.7951
SIM01	Sinovac	Ferulic acid	s__Bifidobacterium_bifidum_fc	-0.0546	0.8044
SIM01	Sinovac	2-Acetylpyrazine	s__Bifidobacterium_bifidum_fc	0.0536	0.8080
SIM01	Sinovac	Mannitol	s__Bifidobacterium_longum_fc	-0.0534	0.8091
SIM01	Sinovac	Ricinoleic acid	s__Bifidobacterium_bifidum_fc	0.0516	0.8153
SIM01	Sinovac	2-Aminoisobutyric acid	s__Bifidobacterium_adolescentis_fc	0.0406	0.8540
SIM01	Sinovac	3-Hydroxyvaleric acid	s__Bifidobacterium_bifidum_fc	-0.0381	0.8628

SIM01	Sinovac	Ricinoleic acid	s__Bifidobacterium_longum_fc	0.0366	0.8691
SIM01	Sinovac	3-Mercaptolactic acid	s__Bifidobacterium_longum_fc	0.0356	0.8727
SIM01	Sinovac	Pyridoxal	s__Bifidobacterium_longum_fc	-0.0356	0.8727
SIM01	Sinovac	Phthalic acid	s__Bifidobacterium_longum_fc	0.0306	0.8905
SIM01	Sinovac	3-Aminobutanoic acid	s__Bifidobacterium_adolescentis_fc	-0.0223	0.9196
SIM01	Sinovac	N2-Acetylornithine	BABBBL_fc	-0.0178	0.9370
SIM01	Sinovac	N2-Acetylornithine	s__Bifidobacterium_longum_fc	-0.0089	0.9694

Table S7. Correlations between the fold change of identified metabolites from baseline and 1-month postvaccination and sVNT levels.

Treatment	Vaccine type	M6 vaccine dose	fold change of identified metabolites	Immune Outcome	Spearman's Rho	*p* value
SIM01	BioNTech	-	Benzoic acid	sVNT levels at 1-month postvaccination	0.2274	0.0484
SIM01	BioNTech	-	myo-Inositol	sVNT levels at 1-month postvaccination	0.0588	0.6134
SIM01	BioNTech	-	N-Formylmethionine	sVNT levels at 1-month postvaccination	-0.0475	0.6833
SIM01	BioNTech	2nd_dose	Benzoic acid	sVNT levels at 6-month postvaccination	-0.1653	0.2186
SIM01	BioNTech	2nd_dose	myo-Inositol	sVNT levels at 6-month postvaccination	-0.0762	0.5724
SIM01	BioNTech	2nd_dose	N-Formylmethionine	sVNT levels at 6-month postvaccination	-0.0761	0.5727
SIM01	BioNTech	3rd_dose	Benzoic acid	sVNT levels at 6-month postvaccination	-0.1782	0.5421
SIM01	BioNTech	3rd_dose	myo-Inositol	sVNT levels at 6-month postvaccination	-0.0418	0.8872
SIM01	BioNTech	3rd_dose	N-Formylmethionine	sVNT levels at 6-month postvaccination	-0.0022	0.994
SIM01	Sinovac	-	Taurocyamine	sVNT levels at 1-month postvaccination	-0.4338	0.0398
SIM01	Sinovac	-	Pyridoxal	sVNT levels at 1-month postvaccination	0.1492	0.4951
SIM01	Sinovac	-	L-Sorbose	sVNT levels at 1-month postvaccination	-0.0613	0.7812
SIM01	Sinovac	-	Ricinoleic acid	sVNT levels at 1-month postvaccination	-0.0079	0.973
SIM01	Sinovac	-	myo-Inositol	sVNT levels at 1-month postvaccination	0.0000	1.0000
SIM01	Sinovac	2nd_dose	L-Sorbose	sVNT levels at 6-month postvaccination	-0.2381	0.5821
SIM01	Sinovac	2nd_dose	Taurocyamine	sVNT levels at 6-month postvaccination	-0.2381	0.5821
SIM01	Sinovac	2nd_dose	Ricinoleic acid	sVNT levels at 6-month postvaccination	0.0952	0.8401

SIM01	Sinovac	2nd_dose	Pyridoxal	sVNT levels at 6-month postvaccination	-0.0476	0.9349
SIM01	Sinovac	2nd_dose	myo-Inositol	sVNT levels at 6-month postvaccination	-0.0238	0.9768
SIM01	Sinovac	3rd_dose	Taurocyamine	sVNT levels at 6-month postvaccination	-0.3333	0.3488
SIM01	Sinovac	3rd_dose	Pyridoxal	sVNT levels at 6-month postvaccination	0.2727	0.4483
SIM01	Sinovac	3rd_dose	L-Sorbose	sVNT levels at 6-month postvaccination	-0.1636	0.6567
SIM01	Sinovac	3rd_dose	myo-Inositol	sVNT levels at 6-month postvaccination	0.0788	0.838
SIM01	Sinovac	3rd_dose	Ricinoleic acid	sVNT levels at 6-month postvaccination	0.0545	0.8916

Table S8. The differential baseline species between the subjects with and without any SIM01 strain engraftment at 3-month postvaccination after 3 months of intervention of SIM01.

feature	metadata	value	coef	stderr	N	N.not.0	*p* value	*q* value
s__Hungatella_hathewayi	SIM01_strain	1	2.9816	0.7138	144	75	0.0001	0.0073
s__Anaerostipes_hadrus	SIM01_strain	1	-3.1637	0.7910	144	117	0.0001	0.0073
s__Erysipelatoclostridium_ramosum	SIM01_strain	1	2.8758	0.7194	144	58	0.0001	0.0073
s__Bifidobacterium_adolescentis	SIM01_strain	1	-3.0548	0.7889	144	72	0.0002	0.0087
s__Bacteroides_galacturonicus	SIM01_strain	1	-2.0176	0.6543	144	49	0.0025	0.0523
s__Clostridium_bolteae_CAG_59	SIM01_strain	1	2.2886	0.7248	144	71	0.0019	0.0523
s__Blautia_coccoides	SIM01_strain	1	1.6546	0.5238	144	28	0.0019	0.0523
s__Ruminococcus_torques	SIM01_strain	1	-1.7810	0.5756	144	81	0.0024	0.0523
s__Dorea_formicigenerans	SIM01_strain	1	-2.0292	0.6182	144	111	0.0013	0.0523
s__Clostridium_symbiosum	SIM01_strain	1	2.0715	0.6624	144	65	0.0021	0.0523
s__Eubacterium_rectale	SIM01_strain	1	-3.0748	1.0445	144	99	0.0038	0.0733
s__Lactobacillus_rogosae	SIM01_strain	1	-1.7477	0.6557	144	50	0.0086	0.1406
s__Eubacterium_ramulus	SIM01_strain	1	-1.9940	0.7430	144	84	0.0081	0.1406
s__Allisonella_histaminiformans	SIM01_strain	1	-1.2465	0.4750	144	40	0.0096	0.1466
s__Dorea_sp_CAG_317	SIM01_strain	1	1.8319	0.7133	144	27	0.0113	0.1498
s__Clostridium_bolteae	SIM01_strain	1	1.9416	0.7531	144	104	0.0109	0.1498
s__Lachnospira_pectinoschiza	SIM01_strain	1	-2.8775	1.1308	144	57	0.0120	0.1505
s__Dorea_longicatena	SIM01_strain	1	-0.9697	0.3994	144	98	0.0164	0.1851
s__Agathobaculum_butyriciproducens	SIM01_strain	1	-0.8961	0.3693	144	131	0.0165	0.1851
s__Firmicutes_bacterium_CAG_83	SIM01_strain	1	-2.1602	0.9049	144	107	0.0183	0.1948
s__Eubacterium_hallii	SIM01_strain	1	-1.2877	0.5507	144	114	0.0208	0.2105
s__Eubacterium_sp_CAG_274	SIM01_strain	1	-1.1551	0.5084	144	45	0.0246	0.2381
